# A Systematic Review of Wearable Sensors for Monitoring Physical Activity

**DOI:** 10.3390/s22020573

**Published:** 2022-01-12

**Authors:** Annica Kristoffersson, Maria Lindén

**Affiliations:** School of Innovation, Design and Engineering, Mälardalen University, Box 883, 721 23 Västerås, Sweden; maria.linden@mdh.se

**Keywords:** wearable sensors, sensor systems, gait and balance, fall detection, physical activity recognition, rehabilitation, neurological diseases, data sets, research shortcomings, user demography

## Abstract

This article reviews the use of wearable sensors for the monitoring of physical activity (PA) for different purposes, including assessment of gait and balance, prevention and/or detection of falls, recognition of various PAs, conduction and assessment of rehabilitation exercises and monitoring of neurological disease progression. The article provides in-depth information on the retrieved articles and discusses study shortcomings related to demographic factors, i.e., age, gender, healthy participants vs patients, and study conditions. It is well known that motion patterns change with age and the onset of illnesses, and that the risk of falling increases with age. Yet, studies including older persons are rare. Gender distribution was not even provided in several studies, and others included only, or a majority of, men. Another shortcoming is that none of the studies were conducted in real-life conditions. Hence, there is still important work to be done in order to increase the usefulness of wearable sensors in these areas. The article highlights flaws in how studies based on previously collected datasets report on study samples and the data collected, which makes the validity and generalizability of those studies low. Exceptions exist, such as the promising recently reported open dataset FallAllD, wherein a longitudinal study with older adults is ongoing.

## 1. Introduction

In this article, we review the use of wearable sensors for monitoring physical activity (PA). The monitoring of PA has a number of different purposes. These include: the assessment of gait and balance, prevention and/or detection of falls, the recognition of various PAs. PA monitoring is also useful for the conduction and assessment of rehabilitation exercises and for the monitoring of neurological disease progression. The monitoring of PAs may seem distant from the monitoring of patients with neurological diseases, but neurological diseases are often characterized by mobility disturbances and the same types of wearable sensors can, and are, being used in both areas. The WHO [1] pinpoints neurological diseases as being one of the greatest threats to public health. Common neurological diseases include dementia, epilepsy, headache disorders, multiple sclerosis, neuroinfections, Parkinson’s disease, stroke and traumatic injuries but also neurological diseases associated with malnutrition and pain.

Undoubtedly, the development and evaluations of advanced wearable sensor systems have the potential to generate algorithms allowing for personalized diagnoses and treatment, i.e., precision health. However, for this to be realized, algorithms need to be developed based on data that is representative to the expected users of the wearable sensor systems. In a previously published systematic review, Kristoffersson and Lindén (2020a) [2] discussed a number of issues that are important to consider when developing and evaluating wearable sensor systems to be used in real-life conditions. These issues include studies conducted with small samples but also those not taking demographic factors, namely age, gender and healthy participants vs patients sufficiently into account. Here, we highlight those factors that are within the scope of this review. Starting with gait, 35% of the non-institutional adults were reported to have an abnormal gait in [3]. Further, age, fall history, mobility impairments, sleep disturbance and neurological disorders are all factors affecting fall risk [4]. Chronic conditions, which become more common in aging persons, affect PA levels, but also activities such as raising from a chair can become demanding [3]. Kristoffersson and Lindén (2020a) [2] write that “the whole motion pattern changes with age and the onset of illnesses related to the human locomotor system” (p. 15). This issue is also discussed in [4] which points out that existing systems have mainly been tested under controlled conditions in laboratory environments and that future work must focus on the conduction of longitudinal studies in real-life conditions on samples including frequent fallers, aging adults and those with neurological disorders [4].

There are also gender differences related to the scope of this article. For example, osteoporosis is more common among women but under-diagnosed among men. The characteristics of osteoporosis are decreased bone mass density and disrupted normal trabecular architecture reducing bone strength [5]. While increasing the risk of fractures after a fall, symptoms may remain unnoticed until a fracture has already occurred [6]. Not only that, but, changes in bone mass density and bone strength are factors increasing the fall risk among patients with osteoporosis. The fear of falling, particularly among women, is associated with more falls [7]. Confidence in balance is also reported as being related to balance and mobility among older women [8]. Another illness associated with falls is thoracic kyphosis, an abnormal convex curvature of the spine at chest height. The illness is more common among older women than men due to losses in estrogen levels [9]. Women with thoracic kyphosis were found to be more likely to have had a recent fall in [7].

Evidently, healthy participants and patients differ in many aspects. Therefore, the inclusion of patients in evaluations of wearable systems is a necessity for increasing the validity of these systems. In addition, studies must be conducted with sufficiently large samples for achieving generalizable results.

The remainder of this article is organized as follows. Section 2 provides a summarized description of the systematic method used for selecting articles from the two literature searches. Section 3 reports on terminology aimed to facilitate the reading of this article. Thereafter, Section 4 reports on all articles relating to fall prevention. This is a complex issue, therefore the section includes articles on gait and balance (Section 4.1), fall detection (Section 4.2) and physical activity recognition (Section 4.3). Section 5 reports on rehabilitation articles and Section 6 on neurological diseases articles. Section 7 reports on the included articles not fitting directly into any of the aforementioned sections. A considerable number of notes are deliberately used within Section 4, Section 5, Section 6 and Section 7. There are multiple reasons for this; to facilitate the reading of this article and the identification of suitable methods that are already being used in different contexts. Section 8 provides a discussion and concluding remarks on the material analyzed in this article.

## 2. Methodology

Previously, Kristoffersson and Lindén (2020a) [2] performed and published a systematic review on the use of wearable body sensors for health monitoring. The original article, which covered a large field of health monitoring, provided a qualitative synthesis of sociodemographic and research-methodological aspects of 73 articles. These articles were retrieved during a literature search conducted in April 2019. The search was repeated on 6 August 2020 and resulted in the retrieval of 31 additional articles fulfilling the original inclusion criteria. Broadly speaking, these articles can be divided into two areas: one being the monitoring and prevention of noncommunicable diseases (45 articles), and one being the monitoring of physical activity and neurological diseases (54 articles). The present systematic review focuses solely on the articles related to the monitoring of physical activity and neurological diseases and provides a deep analysis of these articles.

Following the requirements of MDPI Sensors, the literature searches were systematic and followed the PRISMA guidelines [10]. Search phrases were selected based on the main research question “How are wearable sensors used for health monitoring?” Seven databases were used: Web of Science Core Collection, MEDLINE, Scopus, ScienceDirect, Academic Search Elite, ACM Digital Library and IEEE Xplore. Web of Science Core Collection itself included six indices: SCI-EXPANDED, SSCI, A&HCI, CPCI-S, SPCI-SSH and ESCI. The search phrases varied slightly between databases but the same search phrases were used, both in April 2019 and August 2020. No time limit for the publications were set in the April 2019 search, the August 2020 search was limited to publications from January 2019 to August 2020. An overview of search phrases, hits, the article selection process and distribution of articles is provided in Kristoffersson and Lindén (2020b) [11], which focuses on the use of wearable sensors for monitoring and preventing noncommunicable diseases.

### 2.1. Selection of Articles for This Review

This article presents more in-depth information on a subset of the original 104 articles. Narrowing down the scope of the review to articles on the use of wearable sensors for monitoring physical activity and neurological diseases, articles falling into categories not relating to physical activity monitoring were out of scope. A few articles included in the article category “Additional” in [2] were excluded in this article as it does not report on studies already conducted but only studies to come.

### 2.2. Articles Included in This Review

In this article, we have divided the articles into six article categories: gait and balance (6 articles), fall detection (10), physical activity recognition (12), rehabilitation (10), neurological diseases (9), and additional (7). The category “gait and fall” used in [2] was divided into two separate categories: “gait and balance” and “fall detection”. Further, a few articles in [2] were re-categorized in this article. In total, this review includes 54 articles.

The 54 articles were published between 2010 and 2020. The distribution per year was the following: 2010 (1 article), 2011 (1), 2012 (5), 2013 (2), 2014 (2), 2015 (6), 2016 (4), 2017 (6), 2018 (8), 2019 (14) and 5 articles were published and indexed prior to 6 August 2020. Five articles [12,13,14,15,16] were based on data from ≥1 previously published datasets [17,18,19,20,21,22,23,24,25,26]. These five articles [12,13,14,15,16] were published in 2017 or later and were found to report on the datasets’ demographics and sensors/activities to varying degrees. We therefore report on these articles within Section 4, Section 5, Section 6 and Section 7. Appendix A provides information provided in articles based on datasets [12,13,14,15,16] in Table A1 and the original datasets [17,18,19,20,21,22,23,24,25,26] in Table A2.

In addition, we have conducted follow up searches if the included articles reported on were ongoing or were planned studies. These searches resulted in an additional four included articles. Three of them, which are discussed in Section 4.2, were published during 2019–2021. Interestingly, one of them [27] reports on a new dataset that is also included in Table A2 in Appendix A. Another article, from 2014, is discussed in Section 5.

## 3. Abbreviations and Terminology

To facilitate the reading, this section introduces abbreviations and terminology commonly used within this article. The majority of works use sensors measuring PA, i.e., inertial measurement units (IMUs). For readability, we use the notations accelerometer, 6D IMU and 9D IMU in the remainder of this paper. The notation 6D IMU is used for sensors including a 3D accelerometer and a 3D gyroscope. The notation 9D IMU is used for sensors that also include a 3D magnetometer. A few works use sensors measuring vital signs, such as heart rate (HR), peripheral oxygen saturation (SpO_2_) and respiration rate (RR). Other sensors included are electrocardiography (ECG), electrodermal activity (EDA), electromyography (EMG), energy expenditure (EE), finger tapping, inter-beat interval, photoplethosmography (PPG), pressure, skin temperature (ST) and surface EMG (sEMG). A few works report on the use of motion capture systems such as Vicon^1^ and Microsoft Kinect [28] for validation.

Several common tests for assessing gait and balance are used within the included articles. These include the: sit-to-stand (S2S) test, six-minute walk test (6MWT), 30-s chair stand (30SCS) test, and the Timed Up and Go (TUG) test. A number of works adopt machine learning algorithms, these include: artificial neural network (ANN), bagged tree, convolutional neural network (CNN), decision tree (DT), dynamic time warping (DTW), hidden Markov model (HMM), k-nearest neighbors (kNN), linear discriminate analysis (LDA), naïve Bayes (NB), nearest neighbor with DTW (NN-DTW), random forest (RF), recurrent convolutional neural network (RCNN), support vector machine (SVM), support vector machine recursive feature extraction (SVM-RFE), support vector regression (SVR), time kNN, weighted-SVM (W-SVM) and wireless kNN.

## 4. Fall Prevention

Falls have been pointed out as the second leading cause of accidental or unintentional injury deaths worldwide by the WHO [29] who reports an estimated 646,000 fatal falls per year. Only traffic incidents result in more deaths by unintentional injury. The death rate after a fall is highest among adults aged ≥60. Studying the number of falls leading to death, adults aged ≥65 suffer the greatest number of falls. The majority of the non-fatal 37.3 million yearly falls require medical attention. Together, they are responsible for a loss of more than 17 million disability-adjusted life years (DALYs). Needless to say, the financial costs resulting from fall-related injuries are high. It is reported that the average hospital cost for a fall injury exceeds $30,000 [4]. Preventing falls is important not only from an economical perspective but also for the well-being of individuals. Being afraid of falling is associated with the avoidance of activities in daily living (ADLs), a reduction in performed PAs, depression, the perception of a lower quality of life and falls [30]. Rajagopalan et al. [4] reviewed the state-of-the-art (SoA) in fall detection and prediction systems and found that existing systems focus mainly on detecting falls. There is a need for monitoring systems that can reduce the number of falls and improve quality of life and safety for those who have fallen or are afraid of falling. Fall prediction and prevention systems are important [4]. *We argue that reliable fall prediction methods are necessary if we are to prevent falls.*

The WHO [29] outlines different measures for identifying and reducing the risk of falling, these include: screening for fall risk within living environments, clinical interventions (e.g., medication reviews, low blood pressure treatment, recommendations on vitamin intake, and correction of visual impairment), home assessment and modification to reduce risk, the prescription of assistive devices addressing physical and/or sensory impairments, muscle and balance training, etc.

Predicting falls is complex. The motion patterns, i.e., gait and balance, changes with age and the onset of illnesses affecting human locomotion. Distinguishing near-fall events and fall events require the ability to discriminate between ADLs and fall-related events, i.e., a wearable sensor system should assist in preventing falls from happening. At the same time, the system should not issue alarms when the user is carrying out normal ADLs. In the remainder of this section, we first present articles related to gait and balance in Section 4.1. Thereafter, we present articles related to fall detection and near-fall detection in Section 4.2 and physical activity recognition in Section 4.3.

### 4.1. Gait and Balance

Six works use wearable sensors for assessing gait and balance. Gait is at focus in [31,32,33] while [34,35] focus on balance. TUG is known to predict older adult’s ability to walk independently and [36] presents work on a mobile real-time TUG test for home use. Table 1 shows that the number of older participants, i.e., adults being ≥65 years, in the included studies is low. Most studies have been conducted with both male and female participants. All studies were conducted with healthy participants, i.e., not with patients. One work [35] provides little information on demographics. Both young and old adults participated in two of the gait studies [32,33], however only [33] compares the two groups. Young participants are used in [31,34,36]. Three studies [34,35,36] have been conducted with less than 10 participants and all studies are observational. As shown in Table 2, there is no consistency in the sensor types used, number of sensors, sensor locations or parameters assessed.

**Figure 1:**
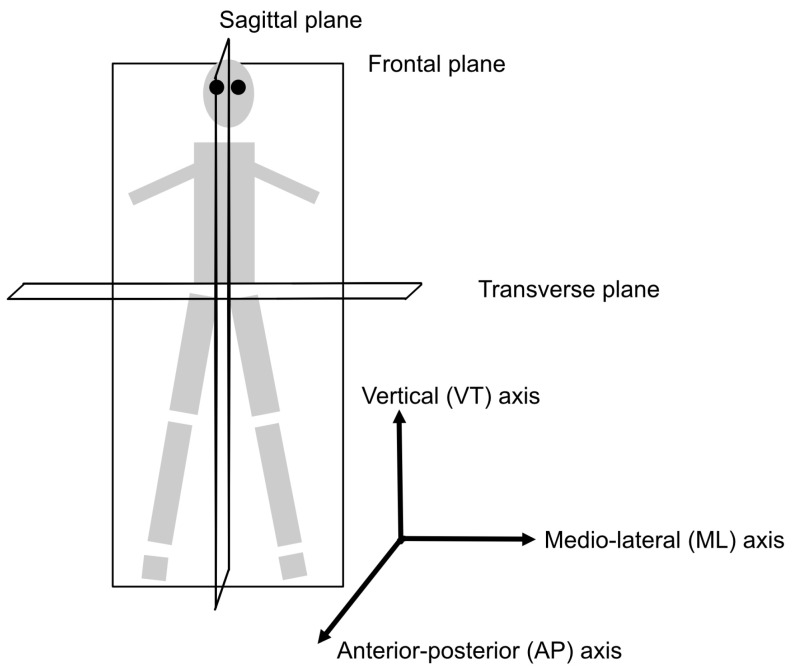
Axes of rotation and anatomical planes. The frontal plane (coronal plane) is dividing the body into anterior and posterior sections, the sagittal plane is dividing the body into left and right sections and the transverse (horizontal) plane is dividing the body into upper and lower sections.

Starting with the works focusing on gait, references [31,32,33] present three different approaches. Aiming at validating the use of an ear-worn accelerometer for gait monitoring, Atallah et al. (2012) [31] asked 34 participants to walk on a force-plate instrumented treadmill at seven different speeds while wearing an accelerometer behind the ear. The features extracted per speed and participant were the zero-crossing, which indicates new gait cycles per axis, the maximal amplitude per stride for each acceleration axis and all possible combinations of axes. Then, multiple linear regression was conducted for assessing the relationship between the maximal amplitude features for all speeds and participants. For comparison, a number of normalized gait parameters derived from the treadmill were used: maximal force, weight-acceptance peak force and impulse. Regarding maximal force, the highest R${}^{2}$ values and lowest estimate of error variance values were obtained for a combination of the VT/AP axes, and for a combination of the VT/AP/ML axes where VT is the vertical axis, AP is the anterior–posterior axis and ML is the medio-lateral axis (the axes are depicted in Figure 1). Regarding weight-acceptance peak force, the highest R${}^{2}$ values were obtained for the same combinations of axes, i.e., the VT/AP and VT/AP/ML axes, whereas the derived R${}^{2}$ values for impulse were lower. Comparing gait cycle parameters predicted by the treadmill and zero-crossings, the errors were higher for the AP and VT axes at lower speeds. Atallah et al. [31] discuss that these speeds may allow for more sway and head movement. The gait cycle parameters derived from accelerometer data were close to the values derived from the treadmill, particularly for the ML axis, with a mean difference of 0.02 s for data within the first standard deviation. It is concluded that the development of regression models using the methodology could provide real-time force-loading features at different speeds.

Godfrey et al. (2014) [32] assess the validity and reliability of using an accelerometer on the lumbar vertebrae (L5) to assess gait. An equal portion of 12 younger and 12 older adults performed two tasks during which they walked five laps along a 25-m oval route at a normal or fast speed. The participants rested for 1 min between the two walking tasks. Additional data was collected using a 7-m-long GaitRite instrumented walkway located along one of the oval’s long-sides (gait characteristics) and a video camera (step counting). Information on step and stride times was estimated based on the extracted initial and final contacts from the accelerometer data collected during laps 2–4 for each speed, according to the method in [37], which is a modification of the method in [38]. Step length was estimated according to the method in [39], after which step velocity was calculated. To compare the accelerometer data with the GaitRite data, only the data segments collected when walking along the GaitRite were extracted. All gait parameters were validated with the younger group. For the older group, similar validity values were achieved for step time and stride times. The estimated step velocity was less accurate for the older group but acceptable according to [40]. *Future work on replicating the full gait model [41] and conducting studies with healthy older adults is reported but we have not found such work.*

Zhong et al. (2019) [33] explored differences in gait patterns between 28 healthy younger and 28 older adults. The 56 participants wore four bracelets equipped with 9D IMUs on their ankles and wrists while walking ten times along a 14-m-long corridor at three self-selected paces: slow, normal and fast. Tape indicated the start and end points and only the middle 10 m for the third and fifth walking trial at each walking pace were used for assessing gait patterns. Gait speed, stride frequency, average stride length, stride regularity, stride time variability and magnitude of acceleration were extracted. In total, the analysis included 336 trials (56 people performing two walking trials at three different speeds). The magnitude of the acceleration, i.e., the root-mean-square (RMS) acceleration was found to increase significantly when the walking pace increased. The RMS accelerations along the AP and ML directions (see Figure 1) were significantly lower for the older group. There was no significant difference between the groups when comparing stride regularity and variability. Technology acceptance was assessed using a 15-item questionnaire. There were significant differences (ρ<0.05) between the older and younger adults’ responses to seven items. The older adults found the “ability to learn how to use the bracelet” and “family support” more important. They also found items relating to the risk of harming the body, measurement accuracy, costs and protecting the privacy of personal data less important than the younger adults. In addition, the older adults were less concerned with the bracelet being inconspicuous, i.e., not clearly visible or attracting attention. The evaluation resulted in a number of possible enhancements: provision of feedback on the bracelet display, access to a gait analysis report and the possibility of sharing data with family members.

Moving on to the works focusing on balance, Paiman et al. (2016) [34] reasoned that knowledge of a user’s current state of balance is necessary to actively prevent falls and to mitigate their consequences. In their work, Paiman et al. [34] proposed to augment collected data with an online observer containing a combination of validated concepts for modelling human gait. The observer’s performance was evaluated by two young people who walked on a treadmill for ≥10 s while wearing a 6D IMU on the back of the waist in four different ways: (1) normal walking at low speed and free arms, (2) normal-speed walking, (3) high-speed walking and (4) normal-speed walking with arms folded at chest height. A motion capture system with five 3D cameras and reflective markers attached to the body from Qualisys was used for tracking movements. Markers were attached to the center of mass (CoM), shoulder joints, hip joints and feet. The participants had difficulties to stand still while the system recorded initial angles. This caused the angle to vary approx. 3${}^{\circ }$ and the calculated error was fed into the observer. Paiman et al. [34] write that the observer could estimate human walking if the system is properly tuned and if instances of foot contact can be estimated correctly. Further, with the virtual pendulum model combined with the virtual pivot concept, the extrapolated CoM and an additive unscented Kalman filter could be used in predictions of human balance. *However, the study was performed with only two young participants.* In another work, Tino et al. (2011) [35] focus on improving body balance by providing vibrotactile feedback. The system contains an accelerometer and vibrating gloves. The accelerometer is worn between the lower shoulder blades and feedback is provided if the CoM has shifted. *Insufficient information is reported on the evaluation.*

Aiming towards a stroke rehabilitation system for home use, Williams et al. (2015) [36] present work on a mobile version of the TUG test, in which a person sits on a chair, stands up, walks 3 m, turns around, returns and sits down again. The mobile TUG test [36] consists of an app and five IMUs. A person conducting the test wears five 9D IMUs: three are attached to the foot, shin and thigh of one leg. One is attached to the torso side and one is attached to the other shin. The mobile TUG test is started when a button is pushed and ends automatically when the person is seated again. The app provides information on when to turn, the person’s position, distance walked, time elapsed and walking velocity. During the 3-m walk, stride length (SL) is calculated using the law of cosines. i.e., taking the maximum angle between the legs α and the length of the right leg (L) as input, α is the sum of the two shin angles while the person is walking. The shin angle is 0 when standing straight. Hence, SL is calculated by solving Equation (1).

(1) $$SL=\sqrt{2L^{2}-2L^{2}\cos\alpha }$$

**Figure 2:**
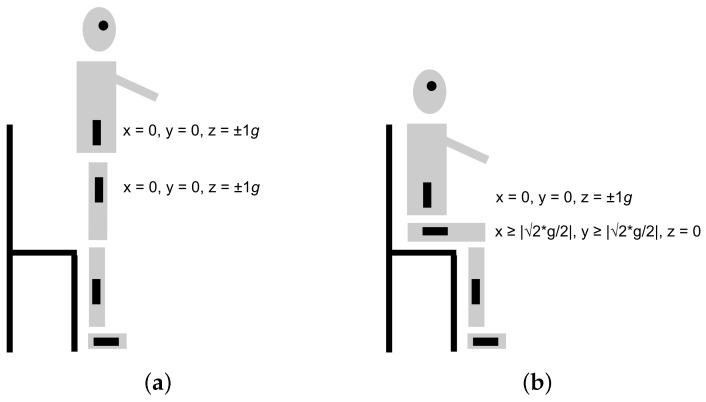
TUG: accelerometer data for thigh and torso in the standing and sitting positions. (**a**) Sensor values when standing. Both sensors are in the vertical position. (**b**) Sensor values when sitting. The thigh sensor is in the horizontal position.

To determine that the person is seated again, data from the two IMUs attached to the torso and thigh are used. If an IMU is in a vertical position, its’ accelerometer value on the *z*-axis is ±1g while the values on the x and y axes are 0. If an IMU is in a horizontal position, the value on the *z*-axis is 0 and the absolute value for the other two axes is $\frac{\sqrt{2}}{2}g$ or higher. A person is sitting if the thigh’s IMU is horizontal and the torso’s IMU is vertical. The x, y and z values for the two accelerometers in the standing and sitting positions are depicted in Figure 2.

Williams et al. [36] report on two evaluations with young people. First, the system’s performance in determining the knee and ankle angles in relation to three prior studies was evaluated by letting one person conduct the mobile TUG test. The knee and ankle angles were detected using the VICON motion analysis system and the mobile TUG app. The app uses the foot and shin IMUs to calculate the ankle angle, and the shin and thigh IMUs to calculate the knee angle. The root-mean-square error (RMSE) of the knee angle for the TUG system in relation to the VICON motion analysis system was found to be lower than in their reference studies. Second, the app’s performance in estimating time and walked distance was evaluated by asking five participants to perform the mobile TUG test five times in a setting wherein the 3-m distance was marked on the floor. They were observed by a physiotherapist who used a stopwatch and by another person who pushed the start button in the app. The physiotherapist told the participants to start and both observers pushed their start buttons when the participants were not in contact with the chair. The physiotherapist pushed the stopwatch button when the participants were in contact with the chair again. The RMSE of time was 0.907 s, i.e., lower than the standard error for TUG, 1.14 s. The RMSE of distance walked was 1.036 m. There were approx. five steps per TUG test. i.e., the RMSE was approx. 0.21 m per stride.

*In this section, we report on six studies. Despite the fact that motion patterns change with age and the onset of illnesses, none of them included both gait and balance. Studies combining these factors would be of interest. Two studies report on the results with older adults. Worth noticing is that step velocity was less accurate among older adults than young adults in [32], and that the magnitude of the acceleration in the AP and ML directions was significantly lower among older adults than young adults when asked to increase the speed in [33]. Therefore, a shortcoming with the remaining four studies is their lack of validation with older adults, i.e., the ones with an increased risk of falling. Another shortcoming with the studies is that none of them were conducted in real-life conditions, a need pointed out in both [2] and [4]*.

### 4.2. Fall Detection

In this section, we report on seven works in which the data was collected by the authors [42,43,44,45,46,47,48]. Two additional works [12,13] used datasets as the basis for their work. In addition, we report on [49] although it is unclear how the system was evaluated. One work focuses solely on fall detection [12]. Six works discriminate between falls and ADLs [13,42,43,44,45,49]. Four works focus on near-fall detection [44,46,47,48]. Three works select optimal sensor locations [13,47,48], however only [48] provides a clear motivation for their selection. One work [13] adds a risk category parameter and classifies subjects into the categories of low-risk, medium-risk and high-risk of falling. As shown in Table 3, only [43] was conducted with older participants or patients. This work also strove to detect abnormal HR. Four works [42,45,46,47] provide information on the participants’ gender. We note that only [42,45] report on the inclusion of female participants. All studies were observational. Only [43] was conducted with more than 20 participants, and three studies [45,47,48] had less than ten participants. There is no consistency in the sensor types used, number of sensors or sensor locations (see Table 4).

**Figure 3:**
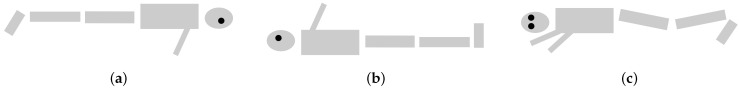
Illustration of people having fallen forward, backward or sideways: (**a**) fallen forward; (**b**) fallen backwards; (**c**) fallen side-ways.

Starting at the lowest level of complexity, i.e., detecting falls which can occur forward, backward and sideways, as depicted in Figure 3, Boutellaa et al. (2019) [12] used data from two datasets^2^ in their work on a covariance matrix for detecting falls. In a first step, one covariance matrix per dataset was built using the variance of each raw sensor signal and the covariance of each sensor signal pair, i.e., the linear association between two sensor signals. Second, data from fused sensors were entered into the matrices. For the CogentLabs dataset, the fused matrix included 15 rows, i.e., one for each possible sensor combinations (the accelerometers, the gyroscopes, six pairs of sensors, four triplets of sensors, and one quadruple of sensors). The corresponding fused matrix for the DLR dataset included seven rows. Third, considering that the datasets include data collected during different activities, three metrics for measuring dissimilarities were applied: Euclidian metric, affine-invariant Riemannian metric (AIRM) and log-Euclidian metric. Accuracy and F-score per row and method were calculated. Analyzing the individual sensors’ ability to detect falls, the data from accelerometers resulted in a high accuracy (>90%) and F-score for both datasets, regardless of sensor location. An even higher accuracy and F-score was retrieved for the magnetometer data in the DLR dataset. Regarding sensor fusion into sensor pairs, the reliability in terms of the F-score was generally not altered regardless of method. Applying the Euclidean metric on the pair of accelerometers, and the log-Euclidean metric on one of the accelerometer-gyroscope pairs in the CogentLabs dataset provided a higher accuracy (92.06 and 92.24% respectively) than data from single sensors. An even higher accuracy (98.31%) was achieved by applying the log-Euclidean metric on the DLR dataset. When fusing data of more than two sensors, the highest accuracy (92.51%) was retrieved by applying AIRM on the quadruple of sensors in the CogentLabs dataset. Comparing to SoA machine learning algorithms, Boutellaa et al. [12] found that their method outperformed other methods.

Moving on to the works focusing on discriminating between falls and ADLs [13,42,43,49], Liang et al. (2018) [42] evaluate a system aimed to provide assistance after the occurrence of a fall. To test the reliability, 18 participants wore a 6D IMU on their waist while falling backwards and performing four ADLs^3^. Each participant performed 20 falls and the four ADLs five times each. Then a subset of the collected data was used to train a HMM and HMM-based SVM model that was used for determining the maximum separation boundary between falls and ADLs. The evaluation indicated that the model’s accuracy, sensitivity and specificity in discriminating between falls and ADLs exceeded 93%.

Fakhrulddin and Gharghan (2019) [43] proposed an autonomous wireless health monitoring system for which they developed algorithms to discriminate between ADLs and falls, and to detect abnormal HR. The fall detection algorithm (TB-AIC) combined an acceleration-threshold (Ath) with an activity/inactivity function (i.e., time threshold—Tth). The acceleration in all directions decreases to near zero after a fall. The signal magnitude vector (SMV, see Equation (2)) is compared with the Ath.

(2) $$SMV=\sqrt{A_{x}^{2}+A_{y}^{2}+A_{y}^{2}}$$

Then, the acceleration values are assessed until Tth, e.g., 20 s. If SMV is unaltered after the 20 s have passed, the TB-AIC algorithm determines that a fall has occurred. To evaluate the algorithm, two healthy young adults and two older adults on medication performed seven different ADLS and falls according to a pre-defined script while wearing an accelerometer on the upper left arm. The script, which consisted of seven sub-experiments was repeated four times by each participant. First, the participants did an ADL (e.g., walking) for 1 min after which they deliberately fell. The sub-experiment was completed 1 min after the fall. The participants then continued with the remaining six sub-experiments. During the experiments, a researcher manually recorded the results from each sub-experiment, including the performance of the accelerometer and whether classification was correct. In total, 224 samples were collected from the four participants, 112 ADLs and 112 falls. One ADL (running down the stairs) was falsely detected as a fall. In addition, one fall, from being seated on the bed to the ground, was not detected. Fakhrulddin and Gharghan [43] reason that the distance was small and that the participant used the arms to break the fall by instinct. *We note that this is also likely to occur instinctively in real-life situations.* Accuracy, sensitivity and specificity for the TB-AIC algorithm all exceeded 99%. The algorithm developed for detecting abnormal HR was evaluated in an experiment with 12 healthy young adults and 12 older adults on medication. The young adults jogged for 3 min while the older adults walked for 3 min while wearing a PPG sensor on their upper arm and a pulse oximeter. The HR estimated from the PPG and the SpO_2_ were statistically compared and there was a good agreement between the two measures. The results reveal that the older adults’ HR was lower than the younger adults’ HR during the activities. This was due to the medication but nevertheless a factor that needs to be taken into account when detecting abnormal HR. *While not discussed in the future work of [43], we find the approach of combining data from the PPG and accelerometers interesting when discriminating between ADLs and falls. Further, a gender difference to consider when using HR as a measure is that cardiac rehabilitation after acute myocardial infarction is both underused and under-prescribed among women despite being recommended in evidence-based guidelines according to [50], which outlines a number of socio-demographic factors associated with lower use of cardiac rehabilitation.*

Information on a system aimed to provide healthcare and fall-detection services is presented by Ghazal et al. (2015) [49]. For fall detection, authors propose using an ANN and a smartwatch’s accelerometer and gyroscope. An alarm should be sent if a fall is detected. For each axis and sensor, a set of unnormalized readings was extracted at a window size of 512. After normalizing the data and noise removal, all filtered windows were merged into a feature vector. The vector’s size was reduced through resampling, then data was fed into a three-layer cascade feed-forward network with 25 neurons per layer. The overall accuracy for activity classification for the non-fall activities was 93.33%. Comparing the performance with DTW, authors found that DTW had a lower fall detection accuracy, which suggests that classification can be improved using the proposed algorithm [49]. *The authors claim to have evaluated the performance by simulating fall events and other activities, however the number of participants is not clearly presented.*

Ramachandran et al. (2018) [13] conducted a literature study resulting in the identification of 23 biological risk factors for falling that were applied to a simulated dataset with 10,000 entries. The odds ratio for each factor was used to derive a weighted normalized score per subject in the dataset. Based on the score, each subject was classified into one of three fall risk categories: high-risk, medium-risk or low-risk. Thereafter, the UMAFall dataset^4^ was used to assess the variation in detection accuracy with and without classifying each subject into a risk category. Then, data was preprocessed in terms of the vector magnitude of acceleration and angular velocity. In a first evaluation phase, the performance of the ANN, kNN, NB and SVM classifiers in identifying forward, backward and lateral falls as well as the ADLs walking, bending, jogging, running, sitting and lying were evaluated. It was concluded that kNN performed better in this phase. In a second phase, a risk category parameter was added to the already existing feature set of data. Thereafter, the classification performance using only data from the wrist sensor was evaluated. Again, kNN performed best. In a follow up work, Ramachandran et al. (2019, 2020) [51,52] explored the effect on performance of combining IMU and HR data in fall detection. A labelled dataset was created by asking 10 young healthy participants (*gender not reported*) to perform 14 ADLs and six different falls, twice, while wearing a smartwatch. RF, which was added as an additional classifier in [51], performed best and the performance increased when adding HR as a feature. Using the same dataset [52] replaced SVM with XGBoost, RF outperformed also XGBoost. *We wish to stress here that no motivation to the choice of adding the RF classifier is provided and statistical tests for differences are not presented. Motivation for replacing SVM with XGBoost in [52] is also lacking. We find it peculiar that SVM was replaced since SVM outperformed NB in the other works [13,51].*

Two works aim to minimize the number of false fall alarms [44,45]. Wu et al. (2019) [44] proposed and evaluated a novel method for detecting falls prior to impact during walking. Thigh and waist angles, and angular velocity around the y-axis were collected from 15 participants wearing two 6D IMUs on the right thigh and waist. Each of them performed 15 walking trials including ≥five gait cycles, 15 fall-like activities^5^, 17 backward falls and 17 forward falls. A fall trial was valid if including ≥two gait cycles. The participants watched fall movies and tried to mimic the falls. After developing a hierarchical classifier based on Fisher discriminant analysis, human activities were classified as: non-fall, backward fall or forward fall. The accuracy in discriminating between activities and falls was ≥95%. In addition, the number of false alarms was significantly reduced when fusing the data from the two sensors (sensitivity: 95.5%, specificity: 97.3%).

Saleh et al. (2019) [45] used a two-step strategy to detect falls and avoid false alarms. The technology used was a wrist-worn accelerometer with a built in embedded algorithm sharing strong similarities with the TB-AIC algorithm for detecting falls [43]. Saleh et al. [45] also compared SMV (see Equation (2)) with a predefined threshold to detect impact. If inactivity was detected, data was sent to a remote server. An experiment was conducted in order to train the SVM and to evaluate the performance in discriminating between real falls and false alarms. Seven young participants performed 19 different ADLs and simulated 35 types of falls. Applying a 10-fold cross validation, it was found that the accuracy was 92.35% for a quadratic-kernel-based SVM and 91.58% for a linear SVM. Then, 121 features (of 12,000 features) were selected using SVM-RFE. Using these features, the accuracy was 100%. To follow up on this work, Saleh et al. (2021) [27] developed an open dataset FallAllD, which contains data from 15 subjects (8 men and 7 women between 21 and 53 years of age) who wore three 9D IMUs combined with a barometer on the neck, waist and/or wrist while performing a selection of 35 possible falls and 44 ADLs. It is shown that using machine learning classifiers is more feasible than deep learning due to lesser energy consumption and that accuracy increases when sampling at 40 Hz instead of 20 Hz. Furthermore, accuracy increases with acceleration measurement range and performance is worse when using data collected at the wrist. However, falling from bed and slowly falling by sliding the back along the wall were difficult to detect regardless of sensor location. It is also discussed that alarms need to be issued even if there is a recovery after a fall. Otherwise, instances of subdural hematoma may remain undetected. It is demonstrated in [27] that performance decreases when taking recovery periods into account. The location resulting in the lowest number of false negatives was the waist. A study with 20 older adults is reported as ongoing.

Moving on to the works focusing on near-fall detection [44,46,47,48], Lee et al. (2015) [46] address the problem of detecting near-falls, falls and ADLs. Eleven students performed a number of falls, near-falls and ADLs while wearing a 9D IMU. The participants performed seven different types of falls found in prior research. Similarly as done in [44], the participants watched movies and tried to mimic the falls witnessed. Each participant made three falls of each type. The near-fall experiments included performing five types of falls^6^ three times. The participants were asked to regain balance instead of falling. The other two fall types were not recorded (faint and stand-to-sit) due to the participants failing to regain balance. All types of falls and near-falls were selected based on prior research. In addition, the participants performed five types of ADLs^7^ three times. Two methods were used to detect falls, one was based on vertical velocity and the other on acceleration. The methods’ performance in discriminating between falls and ADLs was comparable. The vertical velocity based method showed a much higher accuracy (sensitivity: 95.2%, specificity: 97.6%) in discriminating between falls and non-falls (near-falls and ADLs) than the accelerometer-based method (sensitivity: 84.0%, specificity: 85.5%).

The two last works, ref. [47,48] focus on selecting the optimal sensor location for near-fall detection. In [47], Liang et al. (2012) collected data from eight participants wearing four 9D IMUs on the chest, waist, shank and thigh. The participants performed four ADLS once and four types of falls three times each^8^. Liang et al. [47] found using a SMV (see Equation (2)) on accelerometer data to discriminate better between falls and ADLs than did vertical velocity or SMV for gyroscope data. Falling data was separated into three phases: pre-impact, impact and post-impact, where pre-impact is a weightlessness state, impact is when the body hits the ground and post-impact is what happens after the ground hitting. Liang et al. discuss thresholds for altering falls (5 m/s${}^{2}$) and ensuring falls (35 m/s${}^{2}$) providing the pre-impact leadtime of 500 ms. Regarding the selection of optimal sensor location, accelerometry data from the waist- and chest-worn 9D IMUs had higher amplitudes and a lower number of peaks. The waist location was recommended since it lowers the risk of being hurt when falling. *Recall that the waist location also resulted in the lowest number of false negatives when taking the recovery period into account in [27]*. Finally, ref. [47] discusses how data recorded with older adults could differ. It is noted that the recording of data and video monitoring in their living environments would allow for a more true fall analysis.

In another work by Zhao et al. (2012) [48], i.e., the same authors as in [47] and a few additional ones, it was found that the chest is the optimal sensor location for preventing and detecting falls. A system consisting of nine 9D IMUs was implemented and eight participants performed a number of ADLs and falls while wearing the sensors on the chest, fore-waist, left waist, right and left thigh, right and left shank and right and left foot. The ADLs included calibrating the sensors once for each of the actions: stand-sit-stand, walk and stand-sit-lie. Each ADL was conducted three times. The falling activities included one calibration for falling followed by three right sideways falls, three forward falls and three backward falls. Using, again, the SMV equation (see Equation (2)), the authors revised their previous acceleration thresholds for altering falls to 7 m/s${}^{2}$ and 20 m/s${}^{2}$ for ensuring falls. In addition, the critical angles of postural stability during the three types of falls along with pre-impact lead times were studied. The largest postural stability angle (49.9 ± 4.1${}^{\circ }$) was associated with sideways falls. The corresponding postural stability angles for forward falls and backward falls were 23.9 ± 3.3${}^{\circ }$ and 9.9 ± 2.5${}^{\circ }$ respectively. Studying the pre-impact lead times, forward falls were associated with the longest time of body adjustment (329 ± 21) ms. Corresponding pre-impact lead time for sideways falls and backward falls were 265 ± 35 and 257 ± 36) ms. Given these numbers, backward falls are those most difficult to avoid due to the small postural stability angle and shorter pre-impact lead time. *We note that the participants in [46] failed to regain balance when simulating falls while changing from a standing-to-sitting position. The altered CoM during backward falls and falls while sitting down is difficult to restore.*

In another article, Kristoffersson et al. (2021) [53] review the existing evidence for sensor-based fall risk assessment and risk factors for study bias. The review covered 33 studies including ≥10 participants aged ≥60. Approximately 33% of them included at least 100 participants. In 22 of the studies, participants were classified as a faller/non-faller through feature selection. In the remaining 11 studies, classification models were applied. Several sensor features and three classification models were found to vary significantly between fallers and non-fallers. However, too few studies used prospective data, samples were too small and too few participants were fallers. In addition, the used features varied significantly and validation with recommended methods was limited.

*Several of these risk factors have also been identified in this section, ten works about which we have reported on, retrieved during the literature search, and on another three works [27,51,52]. Despite the fact that both age and gender affect fall risk, and potentially, also, injuries after a fall, only a total of 14 older adults (who were aged 61-66) were recruited to the studies. Gender distribution was only provided for five studies, two of them included only men and the remaining ones included a majority of men. None of the studies were conducted in real-life conditions. Hence, the validity and the generalizability of the reported results are low. Worth mentioning, though, is the recently published [27] on the open dataset FallAllD, wherein a longitudinal study with older adults is reported as ongoing.*

### 4.3. Physical Activity Recognition

In this section, we report on nine works [54,55,56,57,58,59,60,61,62] where authors have collected data and three works [14,15,16] based on datasets. Five works focus on classification of PA, postures, and/or PA energy expenditure (PAEE) in controlled environments [54,55,56,57,58]. One work takes this one step further by classifying PAs and estimating PAEE in real-life (2013) [59]. Four works deal with the issue of large-scale monitoring in real-life [14,60,61,62]. The last two focus at detecting bed-exits [15] and life logging to prevent obesity [62]. The observant reader may have noticed the terminology change from ADLs to PAs in this section on physical activity recognition. Table 5 shows that remarkably little information has been provided regarding the participant demographics. Only 5/10 of the conducted studies report age or gender and none of them include older adults (≥65 years). Only one study was conducted with patients. Three works report on sub-studies [58,59,61]. Only [58,59,62] report studies with 20 or more participants. All studies were observational. A variety of different sensor types, number of sensors and sensor locations is reported (see Table 6).

Aiming towards reducing the risk of falling, Awais et al. (2019) [15] propose an internet of things (IoT)-based solution for classifying ADLs. The idea is to detect bed-exits and allow for alerting healthcare professionals such that precautionary actions can be taken. Two datasets [23,24]^9^ were used for this purpose. In [23], 10% of the data was labelled as on-bed, 13% as lying and 87% as off-bed. The proportions were different in [24], wherein 74% of the data was labelled as lying, 24% as on-bed and only 2% as off-bed. During feature extraction, it was noted that a SVM (see Equation (2)) was useful for discriminating between sedentary activities, such as lying and sitting and high intensity activities. Therefore, features extracted from the two datasets included both statistical descriptors and features in the time-frequency domain obtained by the accelerometer and SVM data (see Equation (2)). In addition, the same features were extracted for the received signal strength indicator (RSSI) in [24]. RF, SVM and a W-SVM penalizing the majority represented class were used to classify the data. Studying the classification performance of the data in [23], all classifiers achieved an overall performance of 90% but the performance was lower during sedentary activities for RF and SVM. Performance improved using W-SVM. Awais et al. [15] reason that this is caused by the fact that the activity level is much higher off-bed than during sedentary activities. Studying the classification performance of the data in [24], which included considerably more sedentary behavior than [23], RF performed best overall (88.4%) while W-SVM performed worst. Awais et al. [15] reason that this is in-line with their prior research [63] and that more samples are needed. The recognition of off-bed activities in [24] was approx. 70% for RF and SVM, and 66% for W-SVM. The dataset [24] is affected by noise and packet loss to a larger degree, i.e., the SNR varies with the distance between the RFID’s transmitter and receiver. For detecting bed-exits and alerting staff, the classification needs to be correct and performed in real-time. It is claimed that the proposed system can issue alarms within a 0.1-s delay. Future work outlined includes validating and testing the proposed system in real settings (clinical or residential, *however we have not found such work published yet.*

Continuing with the works focusing on classification of PA, postures and/or PAEE in controlled settings [54,55,56,57,58], Castro et al. (2017) [54] presented an IoT system consisting of a chest-worn band equipped with sensors for measuring HR, RR, ST and accelerometry, a smartphone application (for data reception, classification and visualization) and a cloud (for storage of raw data and recognized activities). The system can provide visual feedback while conducting PAs. Machine learning algorithms were applied on data recorded when three students performed four PAs^10^ in a pre-set order, twice. After feature extraction using a straight line as structural detector and pre-processing using principal component analysis (PCA), classification using C4.5 was correct for 95.83% of the PAs. Future work include a comparison of the performance when using different structural detectors, collection of a larger and more heterogeneous set of training data and the implementation of a classifier in the cloud to overcome current hardware limitations [54], *however we have not found such work.* The work by Rodriguez et al. (2017) [55] has strong similarities with [54] but the number of participants is lacking and jogging is replaced by running.

Rednic et al. (2012) [56] focused on experimental design features (number of sensors, sampling rate, training set size and extracted features) that can influence performance when classifying eight postures^11^ used in explosive ordinance disposal missions. Data was collected from 17 participants using two sets of sensors^12^. The sampling rates for the two systems were 10 and 100 Hz, respectively. After processing the data using a sliding window of 30 samples and a sampling rate of 10 Hz and applying eight different machine classifiers, the classification accuracy for all postures and sensors was cross validated using leave-one-subject-out. It was found to be insensitive down to a reduction to two sensors positioned on the right thigh and calf. The accuracy decreased significantly if only one sensor was used. Then, the most optimal position was the calf. Adequate performance was also achieved when using the hip sensor. The performance was insensitive to sampling rate. Further, the classification accuracy stopped increasing after the inclusion of eight participants in the training set. Studying how the extracted data features affected the classification accuracy, it was found that windowed variance performed best for the postures walking, standing and crawling, whereas exponentially weighted moving average performed best for the postures laying on one side or with face up. The performance in classifying sitting was high regardless of the extracted feature.

Laamarti et al. (2020) [57] presented a digital twin framework for collection of health and well-being data and for motivational interaction with the real twin. Proposing the use of the digital twin for physical exercise motivation and encouragement, a case study was presented. Data was collected from 10 participants performing three PAs^13^ while wearing X73-compliant insoles. Each PA was manually labelled but no further information on the experimental setup was provided. Using a CNN, authors found that the best performance was achieved when the model was trained with data from all participants. Plans for more large-scale and long-term studies were reported.

Aiming at assessing PAEE, Caya et al. (2019) [58] reported on two experiments for training and testing an ANN used for classifying four PAs^14^ associated with different metabolic equivalent task (MET) values. The PAEE was calculated by multiplying the MET value with the duration of the PA. The two experiments included 50 and 30 young adults of both genders but no gender distribution was provided. The participants performed the PAs while wearing a 6D IMU on the wrist. It is not clear how the ANN was trained. For testing the ANN, 30 participants performed each PA for 20 s, and each PA was performed three times. The ANN classified 91.11% of the 360 PAs correctly. The future work suggested included changing sensor locations and classifiers.

Advancing to classifying PAs and estimating PAEE in real-life, Doron et al. (2013) [59] reported the results from an SVELTE project in which a few prototypes and algorithms for classifying nine PAs^15^ were developed. Data was collected in two steps. First, in the laboratory where 65 participants followed a PA path including a total of 23 standardized PAs or postures for 2 h. Each participant spent up to 5 min per PA except for when lying down, where metabolism was estimated over 45 min via indirect calorimetry based on O${}_{2}$ and CO${}_{2}$. The participants wore two accelerometers, up to eight 6D IMUs, a mask for measuring O${}_{2}$ and CO${}_{2}$ and an Actiheart (for measuring EE, HR and inter-beat interval). Information was lacking regarding sensor positions but through sensitivity analysis, ref. [59] determined that the best location for a 6D IMU is the hip. *We note that this was also the second best location in Rednic et al. (2012) [56], which focused on classifying postures used during explosive ordinance disposal missions*. Modelling the PAs with a Gaussian mixture model of two Gaussians, 79.3% of all PAs were correctly identified. However, the detection rate ranged from 55% for sitting to >90% for lying down, walking and running. The real-life experiment was conducted with 20 participants following a PA path in Lyon for approx. 3.5 h while wearing a 6D IMU on the hip, an accelerometer and an Actiheart. A researcher followed the participants and annotated the start and end of each PA. Applying the Gaussian mixture model on the real-life dataset, which included many additional PAs, showed that the algorithm was limited when discriminating between the PAs lying down, slouching and sitting, as well as between the PAs standing and stalling. *It should be noted that [59] provides no definition for what constitutes any of its PAs. Therefore, it is difficult to compare the algorithms’ performance with the aforementioned works [54,56] which appear to present higher classification accuracies for sitting activities.* Ascending stairs was classified as walking 68% or stalling as 20% of the time. Descending stairs was classified as walking during 57% of the time but as running 42% of the time. Standing in a vehicle was identified as stalling 92% of the time while sitting in a vehicle was identified either as slouching, sitting or stalling. *It is unclear if and how information on EE was used in the classification and a database mainly related to EE is reported as being under collection in [59]. A search for this database was unsuccessful.*

Several works [14,60,61,62] dealt with issues related to large-scale monitoring in real-life. Rokni and Ghasemzadeh (2019) [14] argued that the need to retrain machine learning algorithms for new sensor contexts (on-body locations, new users etc) is a major obstacle. Share-n-Learn, where the idea is that new sensor contexts should be automatically detected using a repository of shared expert models, was introduced in [14]. Algorithms should detect dynamic sensor contexts (on-body locations) and activate the most accurate machine learning algorithm for these locations. Such a solution allows for non-static sensor locations. Share-n-Learn trains a gating function using a static sensor together with a dynamic sensor. For each static sensor, there is an expert model for PA recognition. The dynamic sensor is moved around to obtain a repository of expert models, i.e., the static sensor teaches the dynamic sensor (learner) by presenting probability information. The learner compares the probability information with the probabilities provided by the different experts. To demonstrate Share-n-Learn’s effectiveness, data from three datasets 16) performing ADLs while wearing IMUs at different locations. Treating one IMU at a time as the static sensor and the other sensors as dynamic, a repository of experts was built. Assessing the performance of Share-n-Learn when moving a sensor, it was found that Share-n-Learn could accurately detect 68.4% of PAs [14]. *A number of planned future works, including the collection of data in real time, was reported in [14], but we have not found such work. We argue that this is a necessity for overcoming the problem of having to retrain machine learning algorithms for new sensor contexts. We appreciate the intention to develop algorithms allowing for learning from already trained expert models and the possibility to use dynamically moving sensors.*

Xu et al. (2014) [60] presented a system allowing healthcare professionals to subscribe to individualized training programs and to monitor quality and patient compliance. Activities are separated from contexts. Context is the subset of external attributes which characterize an environment or a situation, i.e., meeting is a context whereas sitting in a meeting is not since the latter includes an activity. Different individual activities may be prescribed in each context. Further, ref. [60] introduced scenarios wherein “A scenario is the combination of a context, and a set of activities of interest under that context, with a model for distinguishing the activities” (p. 1017). On a high-level, the system operates in three modes: (1) prescription, (2) training with end users such that the classification system can recognize the context and the end users’ motions and (3) the live monitoring of end users. The system—which comprises an Android tablet app, four 9D IMUs positioned on the mid waist and on the dominant wrist, knee and ankle—obtains sensor data and determines the context and motions using a number of classifiers running as core components in the app. The system’s ability to classify contexts and activities was evaluated by collecting data from 14 participants who spent 30 min in eight different contexts. For each context, they performed a number of PAs for 2–5 min. Then, 30% of the data was used for training four context classifiers: wireless kNN, time kNN, AdaBoost using sound features and a committee approach, performing a linear combination of results from the other three classifiers. The remaining 70% was used for testing. The committee approach performed best. Wireless kNN was not sufficiently accurate in buses and outdoors due to the detection of new access points along bus routes and the simultaneous detection of access points belonging to different outdoor contexts. Time kNN was not sufficiently accurate in a number of contexts due to variations in when contexts were visited by participants. AdaBoost worked well in most contexts, however, misclassifications occurred during long periods of silence and if a bus passed nearby. Thereafter, the performance of the *context-driven personalized activity classifier approach* was evaluated by comparing it against a hierarchical NB using the wireless health institute sensor fusion toolkit (WHISFT). The context-driven approach, in which the system needed to consider only the PAs related to the current context, resulted in a substantially higher classification accuracy and a significantly lower classification time. The number of sensors required to be active can vary between scenarios. Therefore, the approach could lower the energy consumption.

In a more recent work [61], Xu et al. (2016) extend the work by adding the reporting of PAs at different granularity levels including location categories a person can visit, and which PAs can be performed at each location category. Motion trajectories and metrics are computed and can be used for visualizing each PA. A location context detection model using WiFi augmented GPS was also added. The system’s capability of detecting context was evaluated by four participants equipped with a tablet who visited any places matching a number of contexts during a period of 7 h while wearing four 9D IMUs, above the elbow and wrist of the dominant arm and at the top of both shoes. The system performed well in classifying commercial contexts such as restaurants and gyms but less well during residential visits due to the system API’s lack of residential coordinates. WiFi augmented GPS resulted in an improved tablet battery time. The lower body motion tracking algorithm was evaluated in two steps. First, three healthy participants performed three different PAs^17^. Comparing the results against data captured by the Vicon motion capture system, the method [61] was found to reconstruct their lower body motions accurately. Second, five patients with hemiparetic gait after stroke performed the 10 m walking test. The absolute error for distance estimation was lower for the healthy side than for the hemiparetic side. To verify the results, a control group also performed the same test. The upper body motion tracking algorithm was evaluated by six participants performing a number of arm motions. A comparison with the Kinect system for capturing skeleton movements indicated a low error, on average. It was stated that a larger study is needed to ascertain system robustness and to compare the results against golden standards. Further, clinical trials are needed to assess the efficacy of the data provision.

Starting from the standpoint that continuous monitoring of PAs requires energy-efficient strategies, Culman et al. (2020) [62] introduced a change point-based activity monitoring (CPAM) algorithm in which the sampling rate is adapted based on state changes. To test the algorithm, 66 participants wore an Apple Watch on the non-dominant wrist while performing and labelling nine PAs^18^. The watch included an IMU (acceleration, gyroscope, compass), a HR sensor and GPS. It was argued that some PAs are easy to recognize while others, more complex PAs, such as watching TV and listening to a lecture, require location data. The PA recognition accuracy increased from 0.78 to 0.95 when adding location data, and CPAM was found to reduce energy consumption by 74.64%.

Finally, Dobbins et al. (2017) [16] focused on life logging to prevent obesity. Two datasets^19^ are used as input to the system which uses a smartwatch for displaying results. The 16 PAs in the two datasets represent three different PA levels: light, moderate and highly energetic, i.e., PAs associated with different MET values. The approach [16] includes collecting, processing, classifying and visualizing data. Ten different classifiers were evaluated and the approach showed a higher classification accuracy than four prior studies. Future work includes system implementation, a focus group, user acceptance tests and examination of unsupervised methods. *However, we have not found such work.*

*This section has analyzed twelve works. The differences related to age, gender and healthy participants vs patients that were outlined in Section 1 show that PAs may be performed differently depending on the demographic variables. Generally, very little information on demographics is provided. Only one work [15], which is based on a dataset considers older people but without reporting any other sociodemographic information. Therefore, it is unclear how representative the results of the studies in this section are from a broader perspective.*

## 5. Rehabilitation

Ten works report on the use of wearable sensors for rehabilitation purposes. Two works focus at orthopaedic rehabilitation [64,65] and five works focus on stroke rehabilitation [66,67,68,69,70]. Other focus areas include assessing endurance during low back pain rehabilitation to improve trunk muscle status [71], personal classification during lower limb rehabilitation exercises [72] and gait retraining by adjusting the foot progression angle (FPA) [73]. Despite the fact that the studies target rehabilitation, Table 7 shows that only four studies include patients. Looking at the participants’ age, different age spans have been reported. The majority of the studies were observational, only one article [66] was a case-control study. Three studies [66,67,72] had more than 20 participants but [72] included only healthy participants. Three other studies [69,71,73] were conducted with ≤10 participants. All demographics information is lacking in [70]. While several different focus areas are reported, Table 8 shows that all but two articles [70,71] use only accelerometers, 6D IMUs or 9D IMUs in their assessment. The number of sensors used and the sensor locations varied.

Starting with orthopaedic rehabilitation, Argent et al. (2018) [64] explored the opinions of using wearable technology in rehabilitation through one-on-one semi-structured interviews with ten participants representing the clinical disciplines physiotherapy, occupational therapy, clinical nurse specialists, orthopaedic assistants and staff nurse. A prototype of a wearable exercise biofeedback system, consisting of a shank-worn 6D IMU and a tablet Android application, was demonstrated. Clinicians saw a potential for the technology but also design and implementation challenges related to technical accuracy and individual tailoring of rehabilitation. Further evaluation was performed using a mixed method approach in Argent et al. (2019) [65]. Fifteen patients having undergone knee replacement surgery used the system at home for 2 weeks, after which they participated in a semi-structured interview and filled out the System Usability Scale (SUS), and the user version of the Mobile Application Rating Scale (uMARS). To minimize the risk of harming acute patients because of system shortcomings, it was first tested by five post-acute patients, who had undergone their surgeries ≥6 weeks prior to being introduced to the study. Then, 10 acute patients, representing the system’s target group, were recruited 2–3 days post-surgery. They were introduced to the system already before undergoing surgery. The tablet was used to: guide them through exercises, mirror their movements in real-time, count the number of repetitions and provide feedback on the technique at the end of each exercise. The feedback was based on supervised machine learning. Prior to bringing the system home, a 30 min training session including the full set of prescribed rehabilitation exercises was provided. The investigator visited and observed the training twice during the 2-week period of system use, and took notes on system crashes and user errors. SUS scores, which indicates a high usability, was supported by qualitative results. The mean adherence rate was approx. 80% among those reporting a positive user experience. The study [65] resulted in the identification of a number of bugs and inacurracies, but also suggestions for features that could increase user engagement.

Continuing with stroke rehabilitation [66,67,68,69,70], Lee et al. (2018) [66] propose a system for functional recovery of the upper limbs. The system detects if an ADL is consistently performed with the unaffected limb and encourages the use of the affected limb instead. Further, the system assesses the quality of motor performance while the user conducts home-rehabilitation exercises, and uses this knowledge to provide feedback and promote high-quality exercising. The main focus of [66] was the development of algorithms based on data from 20 stroke survivors and a control group with 10 age-matched participants wearing two 6D IMUs on the wrist. The stroke survivors started by performing the Fugl-Meyer Motor Assessment (FMA) [74]. Thereafter, a therapist selected a subset of motor tasks and rehabilitation exercises (≥8 motor tasks and ≥5 exercises) that they should repeat three-to-five times. The control group performed all motor tasks and rehabilitation exercises. All participants were video recorded, after which sensor data was time-synchronized to allow for offline analysis. The therapist used the videos to label the data. First, all data from the unaffected limb of the stroke survivor and both limbs of the controls was labelled as goal directed or non-goal directed. The therapist also used the videos for providing feedback on the performed rehabilitation exercises, i.e., how accurately they were performed and if there were compensatory movements. The label feedback was provided if feedback was provided for situations during which feedback would have been provided during standard rehabilitation. Otherwise, the label no feedback was provided. The labelling was performed for all participants. After preprocessing the ADL data (*we presume this refers to the motor tasks*), a ROC curve was generated using logistic regression and leave-one-subject-out cross validation. The ROC AUC of 87% indicates that there was a strong classification ability between goal-directed and non-goal-directed movements. Feedback on rehabilitation exercises was generated by: (1) pre-processing the time series data, (2) classifying the time-series using NN-DTW, (3) selecting features relevant for determining feedback type through a correlation-based algorithm and (4) classifying the performance into “feedback” or “no feedback” using RF. The system could detect poor performance (F-score = 84.3%) and detect 83.1% of the instances labelled as “feedback”. In addition, ref. [66] reports on focus groups with 17 chronic stroke survivors and 13 occupational therapists. The majority of the occupational therapists found the system adequate and were willing to use it in their clinical practise, and a majority of the stroke survivors would use the system if their occupational therapist recommended them to.

Aiming towards post-stroke continuous monitoring of upper-limb performance, Kim et al. (2019) [67] designed a prototype of a finger-worn ring containing an accelerometer and investigated its efficacy in collecting meaningful information. The use of a ring is motivated by: (1) wrist-worn sensors being insufficient for studying fine-hand movements [75], (2) instrumented gloves being cumbersome to use for real-time monitoring [76] and (3) while a combination of a magnetic ring and a magnetometer on the wrist has been proposed [77], a minimum number of sensors is preferred [78]. Assessment of post-stroke upper-limb performance requires the monitoring of both limbs. Hence, a solution, such as the one proposed in [77], would require four sensors, and Kim et al. [67] argue that this may have a negative effect on adherence. Prior work, ref. [79], indicated a high correlation between self-reported preferred handedness and measures of actual hand usage. A quantitative study with 25 stroke survivors, and two qualitative semi-structured interview studies with the stroke survivors and seven occupational therapists were reported [67]. *In this article, we focus only on the quantitative study, which was conducted in several steps:*

1. Self-reporting using a motor activity log (MAL). For 30 items representing different ADLs, the answer is provided on two scales: amount of use (AoU) and quality of movement (QoM). MAL-AoU focuses on usage of the affected limb while MAL-QoL focuses on the movement quality, i.e., speed and accuracy.
2. Research therapist assessment of impairment level and quality of movement using FMA and a subset of the Functional Ability Scale (FAS) [80].
3. Performing six motor tasks^20^ while wearing two accelerometers on the index fingers and two on the wrists, to allow for comparison. Most tasks were bilateral, others also included unilateral tasks, such as picking up a sock with one hand. The tasks required gross-arm and/or fine-hand motor skills. All tasks, except walking and S2S, were performed twice. Firstly, in the way they would normally perform the task. Secondly, while using the affected limb as much as possible.

The correlation was higher between the finger-worn accelerometer data and MAL-AoU than between the wrist-worn accelerometer and MAL-AoU. The general findings in [67] included: (1) the unaffected limb was more frequently used, (2) the unaffected limb’s intensity was higher than the affected limb’s during bilateral tasks, (3) the use of the unaffected limb increased with task complexity (particularly during the buttoning shirt task) and (4) mild impairment was associated with a more symmetric limb usage than moderate impairment. There was a significant different between the two attempts at putting on socks. Some with a moderate impairment did not succeed at all when asked to perform the task while using the affected limb as much as possible. Kim et al. [67] reason that those with a moderate impairment may not put in this effort if having an mHealth system at home. Their future plans included designing and building a self-monitoring system, and performing lab and field tests. *We have not found such work published yet.*

Still^21^ focusing on using finger-worn accelerometers for hand-use monitoring but using a different sensor, Liu et al. (2019) [68] asked 18 healthy participants to perform 11 motor tasks while wearing four 9D IMUs on the index fingers and wrists. Reflective markers were put on each IMU for Qualisys benchmarking. The tasks, which required different levels of fine-hand and gross-arm movements, were both unilateral and bilateral. The unilateral tasks were performed three times, twice with the dominant hand, and once with the non-dominant hand. There was a high correlation between the first two measures and a significant difference between the second and third measures. Applying SVR and leave-one-subject-out cross validation on the different sensor configurations, it was found that only the finger-worn IMU sensors could capture sufficient information on hand performance. The study limitations highlighted included the use of only 11 motor tasks and a low number of healthy participants. Therefore, the results cannot be generalized to a post-stroke patients or general population [68]. We note that neither [67] nor [68] presented information on the size or weight of the rings, which have different shapes and different sensors/batteries enclosed. Considering the aim of monitoring gross and fine hand use among post-stroke patients, we note that size and weight are both important to consider. Both these factors may cause problems for the carrier and affect their ability to move their fingers and their willingness to wear the rings.

Timmerman et al. (2010) [69] focused on a technology-supported version of the task-oriented arm training regime (T-TOAT), which decomposes skills into smaller, related and functional components. Patients trained by performing each functional component separately before performing the complete skill. An example is the skill “drinking from a cup”, which can be decomposed into functional components, such as reaching out and grasping. The system used was the Philips Research Stroke Rehabilitation Exerciser [81], which comprises a patient station and a therapist station. The patient station includes three 9D IMUs to be worn on the thorax, upper arm and lower arm, an active exercise board for interaction with interactive objects in the real-world and a PC providing exercises and feedback. The therapist station is used to program exercises tailored to the patient. To evaluate patient motivation, system feasibility and the effects of using the system, data was collected from nine participants who were in a chronic stage after a stroke, i.e., >1 year post stroke and diagnosed with a central paresis of one of the arms/hands [69]. The participants trained regularly for 30 min at the clinic during 8 weeks (i.e., 2 times/day, 4 days/week). An occupational- or physiotherapist assisted the participants in adjusting the system, putting on the IMUs, initializing the system and answering questions. To study the impact of T-TOAT, three tests were performed at baseline, after 4 and 8 weeks and AT 6 months post training: (1) FMA to assess arm-hand function/activity, (2) the Action Research Arm test (ARA(T)) to assess upper limb activity level and (3) to assess frequency and quality of use of the affected limb. T-TOAT had a significant and long-lasting positive effect on arm-hand performance. Subjective outcomes from Medical Outcomes Study Short Forms (SF-36) and EuroQoL-5D were mixed. The system usability was perceived as good and participants felt intrinsically motivated and competent while using the system. Overall, ref. [69] found the system feasible but to follow up on the study, Timmermans et al. (2014) [82] performed a randomized control study in which 22 chronic stroke patients performed T-TOAT regularly with or without robotic assistance at the same regularity as in [69]. For every day, patients rested in between the two training sessions for approx 30 min–1 h. *Presumably, this occurred also in [69]*. The patients selected ≥2 skills to train at the beginning of the study, after which they were taught the T-TOAT principles and how to perform the skills’ components and, subsequently, the whole of each skill. Both groups received the same training but one group received assistance by a Haptic Master robot (MOOG, NL), which guided the trajectory through haptic feedback. It was found that the robot-assisted group improved the ARA(T) score significantly during the training and that this improvement was maintained 6 months post-training. Both groups improved the MAL score significantly, also this improvement was maintained after 6 months. The FMA score did not change during the training. Regarding SF-36 and EuroQoL-5D, only the control group showed significantly increased scores during the training. However, the SF-36 score was not maintained during the 6 month post training period.

Bisio et al. (2019) [70] focused on lower limb rehabilitation exercises and proposed the SmartPants system for the remote monitoring of post-stroke patients. SmartPants is composed by four 6D IMUs worn on the thighs and shins and two insoles containing four pressure sensors, each of them connected to the shin IMUs through wires. To test the system’s performance in discriminating between five common rehabilitation exercises^22^, data from patients was collected. *However, information on experimental protocol, the number of patients and demographics was lacking.* After data pre-processing and feature extraction, the performance of eight machine learning algorithms was examined. The RF from any multilayer perception algorithms performed best achieving an accuracy of ≥95% for each exercise. Future plans included applying tensor decomposition and regression techniques to estimate load balance using only the 6D IMUs. *We have found no such work yet.*

Assessing muscle status by testing endurance is part of lower back pain rehabilitation. However, practical limitations in determining such measures using traditional procedures may cause inaccurate diagnoses. To overcome these problems, Banos et al. (2015) [71] conducted a case study on mDurance, a system measuring trunk posture and electrical activity produced by the trunk muscles. mDurance provides a summarized report of the training and a number of measures: total duration, endurance ratio, RMS, average rectified value (ARV), maximum voluntary muscle contraction (MVC) values and a session rating (bad, good or perfect). Trunk measurement is based on Madgwick’s algorithm [83] and uses data from two 9D IMUs worn on the lumbar zone (D12-L1 vertebra) and dorsal. The algorithm outputs a quaternion (4D vector) that is translated to Euler angles representing yaw, pitch and roll. Muscle fatigue is estimated through back EMG. RMS, ARV and MVC values are compared between exercise sessions. In the case study, ten participants (eight men, two women) performed three widely-used functional endurance tests twice. First, while being monitored by a healthcare professional who controlled the starting position and the termination of the test according to pre-established criteria, and second while being instructed on the positions by the mDurance system. The three tests were the: Static Trunk Extensor Endurance Test (STEET [84]), Trunk Curl Static Endurance Test (TCSET [85]), and Side Bridge Endurance Test (SBET [86]). The tests were performed in sequence with a recovery time of >1 h between the two versions of each test. *It is unclear if resting was allowed between tests within each version.* The inter-rater reliability between the traditional version and the mDurance version of each test was estimated using: the intraclass correlation coefficient (ICC)(ρ), Cronbach’s α, and the Bland–Altman “limits of agreements” statistic for continuous variables. There was a high inter-rater reliability for all three endurance tests. Further, three physical therapists evaluated the use of both procedures and found mDurance’s ability to provide guidance on positioning more adequate than the traditional positioning based on instructions from visual inspection. mDurance was considered reliable, since the system indicated an appropriate angle for test initiation. The physical therapists were impressed by the precision of the estimated angle and appreciated the real-time EMG data and by the summarized test report. Automatic logging of time and muscle fatigue values was interesting as this would assist the evaluation of patient improvement. Finally, seven independent experts received instructions on how to use mDurance, finalized the tests and filled out a SUS form. The SUS scores indicated a high acceptability, ease of use and confidence but tests with more experts are needed [70].

The barbell squat is an exercise used during lower limb rehabilitation, but naturally occurring deviations between people is an issue with this exercise. Whelan et al. (2017) [72] adopted a personalized classification approach instead of making a global classification. Comparing the two approaches in a study with 55 healthy participants, the personalized classification performed at a higher accuracy. The approach, which requires only one 9D IMU on the left thigh, has a similar accuracy as a multiple-9D-IMU setup. The approach decreased cost while increasing usability.

Finally, Xu et al. (2017) [73] presented a research platform for retraining gait by adjusting the FPA, a measure related to knee pain. The platform included eight 9D IMUs and a central control unit. Each IMU could provide vibrotactile feedback. In an experiment, six older adults received FPA gait retraining. One 9D IMU was attached to each foot and two 9D IMUs, which provided vibrotactile feedback, were attached at the medial and lateral shanks. *It is unclear if and how the two remaining 9D IMUs were used.* The training was performed in three steps: (1) baseline data collection while walking on a treadmill, (2) toe-in gait training through the provision of feedback on the lateral side of the shank if FPA < −1${}^{\circ }$ or on the medial side if FPA > 9${}^{\circ }$ and (3) toe-out gait training through feedback if FPA < −15${}^{\circ }$ or FPA > −5${}^{\circ }$. The participants walked at 1 m/s for 2 min in each step, and prior to each step, they practised walking and adjusting to feedback for 2 min. The participants walked in the no feedback zone during 68.3% of step 2, and during 89.4% of the step 3. The FPA was highest during step 2 (toe-in gait training).

*This section has reported on nine works retrieved during the literature search and one additional randomized control study with chronic stroke patients [82]. Despite the focus on technology-supported rehabilitation, only five of these works report on the inclusion of patients [65,66,67,69,82]. One additional study [73] was conducted with older adults. This is a strength considering its focus on retraining gait, and the differences in gait between young and old adults presented in [32,33] in Section 4.1. Too much information is lacking in [70] to assess the applicability of the work. We find that [68,71,72] focus at rehabilitation monitoring. However, the results from experiments performed with their respective target groups are lacking.*

## 6. Neurological Diseases

Nine works are reported in this section. Ref. [87] focuses on discriminating between healthy people and those with tremors, while [88,89,90] use the Unified Parkinson’s Disease Rating scale (UPDRS). UDPRS is a tool for assessing Parkinson’s disease (PD) progression over time through evaluating different motor and functional abilities. Diagnosis of Multiple Sclerosis (MS) is at focus in [91,92], and the remote assessment of motor changes among patients with MS (PwMS) is reported in [93]. We report briefly on [94,95], where Memedi et al. (2018) [94] present a mockup of a system for supporting PD patients in symptom management. The mockup was derived by a user-centered design process involving interviews and observations with 11 PD patients. Sok et al. (2018) [95] present a static classification model augmented with a dynamic state estimation model for improved activity recognition accuracy. The model’s effectiveness was validated by 13 ambulatory patients with incomplete spinal cord injuries who performed six PAs^23^ while wearing an accelerometer on the waist. The model aims to aid physicians in selecting physical and drug therapy for improved patient mobility. *We believe that the model could also provide guidance for physicians focusing on patients with PD or MS.* As shown in Table 9, all studies but [87] included patients. One study [91,92] was a case-control study while the other studies were observational. Three articles lack information on the participants’ age and gender. The age spans in the remaining five articles vary. Table 10 shows that sensor type, number of sensors and sensor locations varied. All of them used IMUs, either accelerometers, 6D IMUs or 9D IMUs. The two articles [89,90] reporting on the cloudUDPRS Android app used a smartphone’s accelerometer and screen as sensors. cloudUDPRS is a Class I: medical device for theclinical assessment of PD.

Starting with the works focused at PD [87,88,89,90], Baraka et al. (2019) [87] proposed the use of an accelerometer and forearm sEMG for monitoring human locomotion and study the feasibility of using such sensors for discriminating between healthy people and those with tremors. The 21 participants, who were healthy and 24–66 years old, were instructed on how to mimic tremors, i.e., a PD symptom. Thereafter, they performed the same task twice while wearing an accelerometer and two sEMGs, once while acting as normal and once while simulating tremors. They walked along a 10-m-long straight line, turned and walked back. Elbows were held at a 90${}^{\circ }$ angle with palms up. To allow for comparison with prior work, two sEMGs were used and sampling rate was set to 1000 Hz. After data pre-processing and feature selection, the feature sets were used to train four machine learning classifiers: DT, kNN, LDA and SVM. In addition, adaboosting and bagging were employed to build ensembles of DT classifiers, i.e., classifiers combining predictions of several classifiers. Four different scenarios were used during classification: (1) sEMG on biceps brachii muscle, (2) sEMG on forearm (as proposed by Loconsole et al. in 2019 [96]), (3) sEMG on biceps brachii muscle combined with an accelerometer on the palmar side of the right wrist (as proposed by Rissanen et al. in 2008 [97]) and (4) Baraka et al.’s [87] proposed set of a forearm sEMG and an accelerometer on the palmar side of the right wrist. Regarding scenarios 1 and 2, ref. [87] achieved the highest classification accuracy using a bagged tree (biceps bracchii = 93.1% and forearm = 98.8%). Regarding scenario 2, ref. [96] achieved the highest accuracy (92.8% using SVM) Baraka et al. [87] achieved only 96.9% using SVM and the reason for this difference is due to different sampling rates. Regarding scenario 3, ref. [87] achieved the highest accuracy (98.5%) using a bagged tree. The best accuracy (90%) in [97] was achieved using a clustering iterative k-means technique. Finally, regarding scenario 4, ref. [87] again achieved the highest accuracy (99.6%) using a bagged tree. *Hence, the proposed combination of forearm sEMG data and acceleration data from the wrist appears to be feasible for discriminating between healthy people and people simulating tremor. However, no future work using real PD patients was outlined.*

Two tasks in UPDRS are S2S and leg ability (LA). The patient conducts the S2S task with arms crossed and a score of zero is received if succeeding to stand up on the first attempt. A score of four is received if failing more than once and needing help. When conducting the LA task, the patient sits comfortably, stands up and stomps the feet as high and fast as possible. The task is repeated 10 times per leg. To investigate the feasibility of an automatic detection system being able to associate a set of kinematic features to a UDPRS score, Giuberti et al. (2015) [88] conducted an experiment with 24 PD patients who performed the S2S task while wearing a 9D IMU on the chest. A neurologist simultaneously assigned a score between zero ad four to each patient in which non-integer (0.5 increments) scores could be provided. The performances of three machine learning algorithms (the nearest centroid classifier, kNN and SVM) were calculated. The best performance was achieved for kNN = 4. There was a loose correlation between the UDPRS scores for the S2S and LA^24^ tasks, the majority scored zero on the S2S task but higher on the LA task.

The cloudUPDRS app and an evaluation thereof were presented in Stamate et al. [89,90] that were published in 2017 and 2018, respectively. The app includes a test in which 17 individual observations of kinetic, postural and resting tremor for each hand, leg agility and resting tremor for each leg, clockwise/anti-clockwise hand rotation, single/double target finger tapping on left and right side and gait are made. For each observation, patients took a specific position and performed prescribed movements over 60 s. At the end of the test, the PD patient filled out a form asking for information on the latest medication intake and a subset of the 39-item Parkinson’s Disease Questionnaire (PDQ39). Deep learning, using a RCNN approach, was used to ensure that the observations (acceleration and screen use) were recorded when the PD patients performed the test actions correctly. The algorithm performed at 78% accuracy on a dataset including 227 test sessions with 12 patients and nine different phone models. Aiming at increasing user adoption rates, ref. [90] reported methods reducing the observation time to 20 or 40 s. However, the evaluations showed that maintaining the full 60-s recording is necessary.

Gong et al. (2015, 2016) [91,92], who focused on making a holistic assessment of mobility rather than identifying lower or upper body gait features, reported a causality analysis method and an evaluation thereof. The method assesses the coordination between body extremities for identifying subtle mobility impairments that can assist in the diagnosis of MS. A total of 28 PwMS and a control group with 13 healthy adults performed the 6MWT while wearing five 6D IMUs on left/right wrist, left/right ankle and sacrum. The 6MWT was performed several times over three years and there were 121 valid data sessions, 85 of them from PwMS. The test took place in a hallway and included both walking and turning activities. These activities require different coordination strategies for the body extremities, therefore data was segmented after which phase slope index (PSI) was calculated to check for casual influence and direction of influence. A pairwise causality matrix based on the PSI was built for each data segment and significant and non-significant causalities were discriminated by conducting a binary operation on the matrix using the thresholds recommended in [99]. Using the pairwise causality matrix, causality indices (CI)s representing the associations between different body parts were calculated for each data session. Both an overall CI and individual CIs were calculated, e.g., between the sacrum and right wrist. The four individual CIs found to perform best in separating PwMS from healthy controls were: right wrist—sacrum, right ankle—left ankle, right wrist—right ankle and right ankle—left wrist.

Kuusik et al. (2018) presented a motor condition assessment system for home use by PwMS [93]. The system consists of ≥1 9D IMUs, a phone serving as a communication gateway and a server for storing raw data, processing signals and providing a front-end system. The system was evaluated by 51 PwMS following a test battery with several tests in sequence: (1) the Romberg test with eyes and and closed, (2) lower extremities range of motion (ROM) and movement speed (flexibility) during three sequential exercises while seated, (3) jumping height three times and (4) a spacticity test of lower extremity free drop. The 9D IMU data was collected from thorax in test 1, a thigh in tests 2 and 3 and the foot leg in test 4. Baseline data was observed by a physiotherapist. Then, the PwMS performed one measurement per day at home. After a period of use, the tests were performed at an irregular basis. The results showed that the system is sensitive to motor changes for tests 1 and 2, but no results were provided for tests 3 and 4 due to only a few PwMS performing the tests for clinical pertinence.

## 7. Additional

This section reports on seven works not fitting directly into any of the prior sections. They focus on the monitoring of gym activities [100,101,102], the self-calibration of misplaced sensors [103], a smartwatch application for assessing fitness during a 30SCS test [104], exoskeletons [105] and migraine prediction [106]. As shown in Table 11, demographics are poorly reported and the studies were small, in general. Sensor types, number of sensors and sensor locations varied (see Table 12).

Starting with the works focused on monitoring gym activities [100,101,102], Seeger et al. (2012) [100] designed an Android-based system supporting seamless switching between sensor configurations depending on the event. The system includes four sensors (one HR sensor and up to three accelerometers), myHealthAssistant and associated middleware. myHealthAssistant has three modules: daily activities, gym exercises and pulse monitor. “Daily activities” uses an accelerometer attached above the right knee to recognize four PAs^25^. “Gym exercises” uses two additional accelerometers worn on the chest and in a glove to detect weight lifting exercises. The module discriminates between five cardio workouts and 11 weight lifting exercises and calculates repetitions. “Pulse monitor” uses the last series of detected PAs and current HR. An alarm is triggered if the HR does not match the pulse range associated with the PA. myHealthAssistant presents current HR, calorie expenditure, current exercise, repetition counters and details of the workout. The feasibility of the middleware was investigated in a case study where daily and gym activities were recorded in a fitness diary. Daily PAs and HR were monitored during a one-day long test. *Number of participants is lacking.* There were some negative HR peaks due to dry contacts and some high peaks where the HR was out of the range associated with the PA for a few seconds before returning to normal. The peaks resulted in alarms. *No solution to this problem was presented in [100]*. The participants found the sensors comfortable and easy to attach, but they were uncertain where to position the leg accelerometer. A suggested possible solution was to label the sensor with position information. The leg sensor also slipped occasionally during cardio exercises and a rubber strap for attaching the sensor is considered.

Lim et al. (2020) [101] presented the system MuscleSense. Exercising workload during arm strength exercising was estimated using four different regression models. A pilot study with 20 participants was conducted. They were asked to hold and lift three different dumbbells for 3 s while wearing 8-channel sEMGs and 9D IMUs on the upper arm and forearm. Each dumbbell was lifted three times in a round. There were three rounds. Prior to lifting, the participants were asked to estimate the maximum weight they thought they could complete the rounds with. The average max weight was 8.85 kg. The order of the weights in each round was randomized. Resting times were allowed, of 5 s between repetitions, 30 s between weights and 2 min between rounds. If a participant could not finish all repetitions with a weight, this weight and all heavier weights were skipped in subsequent rounds. R${}^{2}$ for all regressions and all feature sets exceeded 0.80 when cross validated using leave-one-repetition out and leave-one-round out techniques, i.e., they provided a good fit. Using Wilcoxon rank-sum test, the lowest RMSE achieved was 0.683 kg. Future work included the refinement of algorithms to distinguish smaller weight differences between training equipment.

Paay et al. (2019) [102] presented a prototype of the Weight-Mate system, a suit equipped with wired 9D IMUs that should provide real-time feedback on how to adjust body positioning during deadlift training. Weight-Mate was designed through cooperative usability testing and included coaches and weightlifters. Several prototypes and evaluations resulting in different sensor setups and feedback instructions were reported. A formative evaluation of the third prototype was performed. Ten participants wore the suit (seven sensors: four on the back and one of the left calf, right thigh and left arm respectively) while performing a normal training session. i.e., the participants added their ordinary weights to the bar and made their ordinary number of sets and repetitions. Immediate feedback regarding trunk alignment “arch your back” was provided and the data was compared to video recordings of the participants. The results reported in [102] are based on discussions with the participants and on video analysis. Most participants found audio feedback useful, however some found it distracting and occasionally incorrect. All participants found the provision of motivating feedback after every third round appropriate. Post sets, the participants could watch animations providing visual and textual feedback; both feedback types were seen as helpful. Five participants improved their technique between the second and third set. Others reported on problems understanding how to adapt their lifting technique with respect to the feedback. Most participants found the suit comfortable but some suits were too large or too small, and one participant had to adjust the technique because of its size. Several negative comments regarded difficulties putting on/taking off the suit and adjusting the sensors. Future work reported include the provision of feedback at a higher granularity, making the suit wireless, and to use a sensor setup allowing for a higher data transmission rate. The aim was also to test the system in a real gym [102]. *We have not found any published follow up work on either [101] or [102] yet.*

Starting from the challenges arising from users’ failure to attach IMU sensors correctly on their bodies, Wu et al. (2013) [103] presented a self-calibration process of sensor misplacement based on repetitive motion signatures to increase system robustness. Three cases of sensor misplacement are outlined: (1) disorientation where the sensor is rotated around the z axis, i.e., not perfectly aligned horizontally, (2) rotational displacement where the sensor is rotated around the x–z plane and (3) linear displacement where there is a translation along the y axis, e.g., sensors mounted at different height. Only the first two cases are handled in [103] (see the paper for further information on the methods used for self-calibration). Two experiments were conducted to evaluate how self-calibration can improve lower-body tracking and step length estimation. In both experiments, participants wore five 9D IMUs (called sensors in this paragraph for readability purposes), four sensors correctly attached to the waist, thighs and calf with their x–y plane aligned to the sagittal plane (see Figure 1) and the y axis with gravity. *Walking instances were recorded but the task is not described.* One sensor was misoriented or rotationally displaced on the right thigh. Two calibration methods were used. For intra-instance calibration, training data and testing data were extracted from the same walking instance. Since this is not the case in real-life situations, also inter-instance calibration, in which training data from the correctly attached sensors during one walking instance was compared with test data from another walking instance, was used. Training and test data were synchronized using DTW. To estimate signal recovery performance between walking instances, a validation set was needed [103]. Therefore, signals from the four correctly attached sensors in one walking instance are used for training. The signal from the misplaced sensor in another walking instance was used for testing, while the signals from the four correctly attached sensors were used for validation. The signals from the misoriented sensor were analyzed on a dataset including 17 walking instances from five participants. The misoriented sensor’s accuracy was comparable to the other sensors’ accuracy. Thereafter, 40 walking instances from seven participants wearing a rotationally displaced sensor (by 45, 90 or 180${}^{\circ }$) were collected and used to assess step length estimation performance. The 45${}^{\circ }$ rotational displacement did not distort the signal severely while the larger displacements degraded the step length estimation performance. However, this degradation could be effectively corrected to approx. 95% step length accuracy using either of the two calibration methods, although intra-instance performed slightly better.

Jovanov et al. (2019) [104] developed an Android smartwatch application for assessing the number of stand-ups and HR during a 30SCS test. A pilot test with 12 participants wearing either a Polar or Fossil smartwatch was conducted. They were told to start and stop the 30SCS test when the smartwatch vibrated. After the test, they rested on a chair. The application could accurately detect 98.8% of the stand-ups. Compared to ECG, the average HR accuracy was 64% for the Polar and 39.2% for the Fossil smartwatch. The accuracy was higher during baseline and recovery.

Jacob et al. (2020) [105] proposed a full-body exoskeleton system for paralyzed people. The exoskeleton has 15 degrees of freedom and brain signals from 16 EEG electrodes are used to control the exoskeleton. Evaluating the system with ten paralyzed persons performing six natural movements, 85% of the human intentions were accurately recognized. *However, we note that the information provided on the validation procedure was lacking information on which movements were performed.*

Finally, aiming towards predicting migraine, Pagán et al. (2016) [106] derived 15 prediction models using grammatical evolution techniques and obtained prediction horizons of approx. 20 min, which allows for the intake of preventative medication. For modelling, two datasets with ECG, EDA, ST and SpO_2_ for patients A and B were recorded. Patient A had 15 migraine events and patient B had 8 migraine events. The grammatical evolution technique can run in real-time and it is possible to implement it on low-power, wireless monitoring devices.

*This section has included very different works. We find the ongoing work promising and exciting. Clearly, these technologies can also be drivers pushing the development of the technology in the other article categories presented in this article.*

## 8. Towards Advanced Wearable Sensor Systems for Personalized Physical Activity Monitoring

We have now provided in-depth information on the included 54 works related to primarily assessing gait and balance, preventing and/or detecting falls, the recognition of physical activities (PA), rehabilitation and neurological diseases. Follow-up searches based on the included works resulted in an additional four works that have also been reported upon in this article.

While advanced wearable sensor systems can be used to generate algorithms supporting precision health, the algorithms need to be based on representative data, i.e., with the expected users of wearable sensor systems. This entails taking demographic factors, such as age, gender and the health and number of patients into account to a sufficient degree, and also conducting studies in real-life conditions and with sufficiently large samples. Further, there is a need to conduct longitudinal studies in real-life conditions with representative samples [4].

Starting with age, approximately one third of the adults in [3] had abnormal gait. Chronic conditions also become more common when aging and may affect the individual PA level. PAs previously perceived as easy to perform, such as raising from a chair, may become demanding [3]. Age is also one of many factors affecting fall risk [4]. Despite these issues, only two of six (33%) works focused on gait and balance in Section 4.1 included old adults. The results of the two works support the need to consider demographic factors, ref. [32] found that step velocity was less accurate among old adults than young adults, while [33] found a significantly lower magnitude of acceleration in two directions (anterior-posterior and medio-lateral) among old adults than young adults. Moving on to the 13 works focusing on fall detection in Section 4.2, only a total of 14 adults ≥60 years of age were included. The trend is similar among the 12 works focusing on physical activity recognition; only one of the datasets [24] used in [15] included older adults (14). This is interesting since it can be expected that the way in which PAs are performed when aging is differing from the way in which younger adults perform the same PAs.

Continuing with gender, Section 1 reported that osteoporosis (decreased bone mass density and reduced bone strength) is more common among women while under-diagnosed among men. While increasing the risk of fractures, the symptoms of osteoporosis may be unnoticed until a fracture has occurred [6]. Another factor affecting fall risk is the fear of falling [7], which is more common among women, and as is confidence in balance [8]. Thoracic kyphosis (abnormal convex spine curvature) is another illness associated with falls that is more common among women due to lowered estrogen levels [9]. Revisiting the two studies reporting on the inclusion of older adults in Section 4.1, only [8] report on the gender of the participants (five men, seven women). As for 14 older adults, in [43] from Section 4.2, gender information is not provided. Information on the gender of the 14 older adults included in the dataset [24] used in [15] in Section 4.3 is lacking. Regarding the works focusing on rehabilitation, only five of nine works report gender, and the gender distributions vary. The situation is similar for the works reported in Section 6, Table 9.

Moving on to the need to include patients and not only healthy participants, Section 4.1, which focuses on gait and balance, reports no studies with patients. One of ten works on fall detection (Section 4.2), and one of twelve works on physical activity recognition (Section 4.3 include patients. Not even the works focusing on rehabilitation (Section 5 report mainly on samples with patients. Only four of nine studies included patients and two studies do not report on whether the sample consisted of patients or healthy participants. The distribution is better in Section 6, which reports on nine works focusing on neurological diseases. Only one of the studies [87] was not performed with patients. The natures of the articles included in Section 7 differ significantly but the two works targeting patients (paralyzed people [105], and patients with migraine [106]) included patients in their studies.

Regarding real-life conditions, very few of the works reported upon in this paper report on studies conducted in real-life conditions. Three of these [59,60,61] focused on PA recognition in real-life. Doron et al. (2013) [59] asked 20 participants to perform PAs over 3.5 h in Lyon, France while wearing a 6D IMU, an accelerometer and an Actiheart. Their Gaussian mixture model, used for classification, was limited when discriminating between several of the recorded PAs. Xu et al. [60] asked 14 participants to spend approx. 30 min in eight different contexts. In each context, they performed a number of PAs for 2–5 min each; the possible PAs varied between contexts. Four 9D IMUs were used for data collection. Classification accuracy increased while classification time decreased when only considering PAs possible in a context. Xu et al. (2016) [60] extended this work by adding WiFI-augmented GPS. Four participants, wearing four 9D IMUs, visited self-selected places in pre-specified contexts over 7 h. Using GPS coordinates, performance was good when discriminating between commercial context but less good in residential contexts. A fourth work [65] focused on home-rehabilitation of patients having undergone knee replacement surgery. Patients wore a 6D IMU while performing prescribed rehabilitation exercises and a system mirrored their movements and provided feedback.

To sum up the discussion on age, gender, patients vs healthy participants and real-life conditions, this paper shown that none of these has, so far, been taken into account to a sufficient degree. It might be argued that the search phrase used to identify included papers was wide, however, other efforts, e.g., Kristoffersson et al.’s (2021) review, which focused on fall risk assessment studies with older adults, have identified study shortcomings, such as the need to use more prospective data, small samples and the use of too few fallers.

As mentioned in Section 1, this article contains a considerable number of notes. From the information provided in these notes, it is clear that there are works discriminating between partly the same types of ADLs/PAs within the different article categories of fall detection (Section 4.2), physical activity recognition (Section 4.3), neurological diseases (Section 6) and additional (Section 7). Clearly, it could be beneficial for researchers to compare their achieved classification accuracies and potentially further develop algorithms originally developed and/or used in another context.

Five of the included works [12,13,14,15,16] were based on data from ≥1 previously published datasets [17,18,19,20,21,22,23,24,25,26]. Interestingly, Table A1 and Table A2 in Appendix A show that there are considerable differences with regard to how the included works and the original datasets report on demographic information and the sensors used. We find it a serious flaw that [12,13,14,15] do not provide full details on participant demographics. The situation is even worse for [16], which even reports on a higher number of participants for one of the datasets than the original dataset [26] does). We also note that one of the datasets itself [21] lacks sociodemographic information. It is surprising that [15] informs us that the dataset [24] included an unknown number of elderly when reporting that the [24] included 14 older adults, aged between 66 and 86 years of age, would have strengthened the work. Obviously, this shortcoming affects the trustworthiness of works based on datasets [12,13,14,15] significantly, since interpretations regarding the previously highlighted factors—age, gender, patient vs healthy participants and real-life conditions—cannot be made.

Continuing with how the works based on datasets report on the sensors used, Ramachandran et al. (2018) [13], who used the dataset [19] in their work on fall detection, used data from two 9D IMUs. The data represented three fall types and six ADLs. We note that no information is provided regarding the fact that [19] included data from two additional 9D IMUs and four additional ADLs. Considering that the work [13] focused on discriminating between falls and ADLs, it is surprising that data from all sensors and ADLs included in [19] was not used and that a motivation for not using this data is lacking.

Moving on to the works focusing on physical activity recognition, Rokni et al. (2019) [14] based their work on three datasets [20,21,22]. The sensor reporting in [14] differ from the original information for all three datasets: (1) [14] reports that the five 9D IMUs were located at different body locations despite the fact that [20] specifies all body locations, (2) [14] use five IMUs of unspecified dimension while [21] included 24 custom acceleration and gyroscope sensors and (3) [14] used five IMUs (3D accelerometer, 2D gyroscope) while [22] included six. The lack of motivation for not using all sensors from any of the three datasets along with the incorrectly provided information on sensor positions and dimensions severely affect the validity of the work presented. Dobbins et al. (2017) [16] based their work on two datasets [25,26]. However, information on the used sensor is incorrectly reported as accelerometers instead of 9D IMUs in [25]. Further, information on sensor positions is lacking despite being provided in [25].

In this article, we have also included an additional dataset [27] that was identified identified during the follow-up searches performed while synthesizing the results. In the FallAllD dataset, 15 subjects wore three 9D IMUs at three different locations while performing a selection of 35 falls and 44 ADLs. The purpose with the data collection was not to use data from all IMUs but to determine the most appropriate location and classification algorithm for detecting falls. Saleh et al. (2021) [27] determined that machine learning is more suitable than deep learning due to limited computational and battery capacity resulting in a low number of sensor features. Reasoning that alarms need to be issued also when recovering after a fall to detect, e.g., subdural hematoma, data from the waist sensor resulted in the lowest number of false negatives. *We are happy to note that [27] informs us that their algorithms are currently being evaluated in real-life conditions by 20 older adults.*

## Figures and Tables

**Table 1:** Participant demographics for studies on gait and balance. - = no information.

Ref.	Research Design	No. of Participants	Age Group	Age Statistics	Male/Female	Patient/Healthy
[31]	observational	34	-	28.22 ± 12.77	21 /13	0/34
[32]	observational	24 (12/12)	20–40	32.5 ± 4.8	7/5	0/12
				65.0 ± 8.8	5/7	0/12
[33]	observational	56 (28/28)	-	24.6 ± 2.7	14/14	0/28
			>55	66.1 ± 5.0	18/10	0/28
[34]	observational	2	28 and 24	-	1/1	0 /2
[35]	observational	3	40–70	-	-	-
[36]	observational	5–6 (1/5)	27	-	1/0	-
			21–36	27	4/1	-

**Table 2:** Study characteristics for studies on gait and balance.

Ref.	Sensor and Amount	Sensor Location	Aim
[31]	1 accelerometer, 1 force-place instrumented treadmill for validation	on the ear	validating the use of an ear-worn accelerometer for monitoring gait
[32]	1 accelerometer, 1 GaitRite instrumented walkway for validation	on the lumbar vertebrae (L5)	validate the use of an accelerometer worn on the lower back for monitoring gait
[33]	4 6D IMUs	on the ankles and wrists	assess and compare younger and older adults’ gait
[34]	1 6D IMU, 1 Qualisys motion capture system for validation	on the back at waist	estimate state of balance
[35]	1 accelerometer	between the lower shoulder blades	improving balance through feedback when center of mass is shifted
[36]	5 9D IMUs	on the foot, thigh, two shins, torso side	mobile TUG test

**Table 3:** Participant demographics for studies on fall detection. - = no information.

Ref.	Research Design	No. of Participants	Age Group	Age Statistics	Male/Female	Patient/Healthy
[42]	observational	18	-	25 ± 3.24	12/6	0/18
[43]	observational	4	2 23–29, 2 61–66	-	-	2/2
		12	12 23–29	-	-	0/12
		12	12 61–66	-	-	12/0
[44]	observational	15	20–27	-	-	0/15
[45]	observational	7	-	young	4/3	-
[46]	observational	11	-	27.6 ± 4.3	11/0	0/11
[47]	observational	8	-	23 ± 3.45	8/0	0/8
[48]	observational	8	-	28.5 ± 4.3	-	0/8

**Table 4:** Study characteristics for studies on fall detection.

Ref.	Sensor and Amount	Sensor Location	Assessment
[42]	1 6D IMU	on the waist	discriminate between falls and ADLs, separation of fall data into three phases: pre-impact, impact and post-impact
[43]	1 accelerometer, 1 PPG, 1 SpO_2_	accelerometer and PPG on upper left arm, SpO_2_ on the finger tip	discriminate between falls and ADLs, detect abnormal HR
[44]	2 6D IMUs	on the waist and right thigh	discriminate between falls and ADLs, classification into: non-fall, backward fall and forward fall, pre-impact fall detection
[45]	1 accelerometer	on the wrist	discriminate between falls and ADLs, machine learning is used to avoid false alarms
[46]	1 9D IMU	on the anterior side of waist	near-fall detection
[47]	4 9D IMUs	on the chest, waist, shank, thigh	discriminate between falls and ADLs, best feature for pre-impact and impact detection of falls
[48]	9 9D IMUs	on the chest, fore-waist, left waist, right and left thigh, right and left shank, right and left foot	optimal placement for preventing/detecting falls, pre-impact thresholds
[49]	1 smartwatch (1 6D IMU)	on the wrist	discriminate between falls and ADLs

**Table 5:** Participant demographics for studies on physical activity recognition. - = no information.

Ref.	Research Design	No. of Participants	Age Group	Age Statistics	Male/Female	Patient/Healthy
[54,55]	observational	3	-	-	-	-
[56]	observational	17	-	-	10/7	-
[57]	observational	10	25–60	-	5/5	-
[58]	observational	50	16–32	-	-	0/50
		30	16–32	-	-	0/30
[59]	observational	65	-	-	-	-
		20	-	-	-	-
[60]	observational	14	-	-	-	-
[61]	observational	4	-	-	-	-
		3	-	-	-	0/3
		5	-	-	-	5/0
		6	-	-	3/3	-
[62]	observational	66	young	-	-	-

**Table 6:** Study characteristics for studies on physical activity recognition.

Ref.	Sensor and Amount	Sensor Location	Assessment
[54,55]	HR, RR, ST, 1 accelerometer	elastic band on chest	discriminate between four PAs
[56]	Class-act: 11 accelerometers, Shimmer: 5 accelerometers	Class-act: symmetrically on the right calves, upper and lower arms, the chest and on the right hip and ankle. Shimmer: right calf, thigh, upper arm, lower arm and chest	classify eight postures
[57]	24 force-sensitive resistor sensors, 2 9D IMUs	X73-compliant insoles	PA life logging
[58]	1 6D IMU	on the wrist	detection of PA and PAEE
[59]	2 accelerometers, up to eight 6D IMUs, EE, HR, inter-beat interval, O${}_{2}$, CO${}_{2}$	a mask on the mouth, other locations unclear	detect PA and posture, PAEE
	1 accelerometer, 1 6D IMU, EE, HR, inter-beat interval	IMU on the hip, other locations unclear	detect PA and posture, PAEE, real-life data PA
[60]	4 9D IMUs	on the dominant wrist, knee, ankle and mid-waist	context classification, context-driven activity classification, real-life PA
[61]	4 9D IMUs, GPS, Vicon motion capture system, Kinect system	above the elbow and wrist on the dominant arm, on the top-front of shoes	context-guided activity classification, real-life PA
[62]	1 Apple Watch	on the non-dominant wrist	reduce energy consumption in real-life PA monitoring

**Table 7:** Participant demographics for studies on rehabilitation. - = no information.

Ref.	Research Design	No. of Participants	Age Group	Age Statistics	Male/Female	Patient/Healthy
[65]	observational	15	-	63 ± 8.32	6/9	15/0
[66]	case-control	20	-	54.4 ± 10.1	-	20/0
		10		53.8 ± 11.4	-	0/10
[67]	observational	25	-	67 ± 12	-	25/0
[68]	observational	18	18–40	-	-	0/18
[69]	observational	9	-	60.7	5/4	9/0
[70]	observational	-	-	-	-	-/0
[71]	observational	10	21–37	-	8/2	-
[72]	observational	55	-	24.21 ± 5.25	37/18	0/55
[73]	observational	6	-	72.5 ± 6.0	3/3	-

**Table 8:** Study characteristics for studies on rehabilitation. * = the system contains eight IMUs but only six IMU locations are specified.

Ref.	Sensor and Amount	Sensor Location	Aim
[65]	1 6D IMU	on the shank	mirror movements during knee rehabilitation exercises
[66]	2 6D IMUs	on the wrists	detect whether an ADL is conducted with the unaffected limb and encouraged the use of the affected limb
[67]	2 prototypes of ring-worn accelerometers, 2 MTw accelerometers	rings on the index fingers, MTws on the wrists	evaluate finger-worn accelerometers efficacy in measuring information relevant for clinicians
[68]	4 Arcus 9D IMUs	2 on fingers, 2 on the wrists	monitor amount of hand-use in ambulatory settings
[69]	3 9D IMUs	on the thorax, upper and lower parts of the affected arm	measure arm/hand function/activity and program exercises tailored to the patient by dividing skills into sub components
[70]	4 6D IMUs, 8 pressure sensors	IMUs on the shins and thighs, pressure sensors in footwear insoles	recognize lower limb rehabilitation exercise
[71]	2 9D IMUs, 1 EMG	IMUs on the lumbar zone (D12-L1 vertebrae and dorsal), EMG on back	measure trunk endurance in back pain rehabilitation
[72]	1 9D IMU	on the left thigh	personalized classification to evaluate exercise technique
[73]	8* 9D IMUs	top of each foot, and two providing vibrotactile feedback on the medial and lateral sides of each shank	measure FPA and provide vibrotactile feedback to adjust FPA

**Table 9:** Participant demographics for studies on neurological diseases. - = no information.

Ref.	Research Design	No. of Participants	Age Group	Age Statistics	Male/Female	Patient/Healthy
[87]	observational	21	24–66	-	18/3	0/21
[88]	observational	24	31–79	65.9 ± 12.3	17/7	24/0
[89,90]	observational	12	-	-	-	12/0
[91,92]	case-control	41 (28/13)	-	40.5 ± 9.4	25%/75%	28/0
			-	39.3 ± 10.3	47%/53%	0/13
[93]	observational	51	-	-	-	51/0
[95]	observational	13	22–50	-	9/4	13/0

**Table 10:** Study characteristics for studies on neurological diseases.

Ref.	Sensor and Amount	Sensor Location	Aim
[87]	1 accelerometer, 2 sEMGs	accelerometer on the palmar side of right wrist and sEMG on both the right biceps brachii muscle and forearm	discriminate between healthy participants and participants simulating tremor
[88]	1 9D IMU, 1 Optoelectronic system for accuracy validation	IMU in velcro strap on the chest. x and y axes aligned to frontal plane of user and spine	investigate the feasibility of an automatic detection system being able to associate a set of kinematic features to a UDPRS score
[89]	smartphone (accelerometer, screen and cloudUDPRS app)	finger tapping: on the screen, tremor and leg; agility: in the palm; gait: in a pocket or belt	clinical unsupervised assessment of motor symptoms of Parkinsons’ building on UDPRS
[90]	see [89]	see [89]	increase user adoption rate by decreasing the recording time during testing
[91,92]	5 6D IMUs	on the left and right wrists, left and right ankles, sacrum	discriminate between healthy and PwMS through causality analysis
[93]	≥1 9D IMUs	on a pendant and on the thigh, foot leg	a motor condition system for home use targeting PwMS
[95]	1 accelerometer	on the waist	develop a model aimed to support physicians’ selection of physical and drug therapy for improved patients’ mobility

**Table 11:** Participant demographics for the additional conducted user studies. - = no information.

Ref.	Research Design	No. of Participants	Age Group	Age Statistics	Male/Female	Patient/Healthy
[100]	-	-	-	-	-	-
[101]	observational	20	19–30	24.1 ± 3.3	10/10	-
[102]	observational	3	23, 27, 29	-	3/0	-
		3	23, 27, 27	-	-	-
		10	18–29	-	9/1	-
[103]	observational	7	-	-	-	-
[104]	observational	12	20–75	39.1 ± 19	6/6	-
[105]	observational	10	-	-	-	10/0
[106]	observational	2	-	-	0/2	2/0

**Table 12:** Study characteristics for the additional conducted user studies.

Ref.	Sensor and Amount	Sensor Location	Aim
[100]	up to 3 accelerometers, HR	HR on the chest, 1 accelerometer above the right knee for daily activities, gym exercises: 2 additional accelerometers on the chest and weight-lifting glove	automatic event-dependent configuration of sensors
[101]	2 Myo: 8-channel sEMG and 9D IMUs	on the upper arm and forearm	detect workload during arm strength exercises
[102]	4 9D IMUs	on cloth on the calf and thigh	provide corrective feedback during deadlifting
	14 9D IMUs	in suit: on each calf, thigh, shoulder, upper arm and forearm, respectively, and four along the trunk	
	7 9D IMUs	in suit: one on the left calf, right thigh and left forearm, respectively, and four on the back	
[103]	5 9D IMUs	on the waist, thighs, calf and incorrectly on the thigh	self-calibration in cases of sensor displacement
[104]	Smartwatches Fossil Gen 4 and Polar M500: HR, acceleration; ECG for accuracy validation	on the wrist	develop a smartwatch application for 30SCS tests to assess fitness level
[105]	EEG	on the skull	develop exoskeleton to provide support paralyzed people
[106]	ST, EDA, ECG, SpO_2_	on the chest and finger	predict migraines

## Data Availability

Not applicable.

## Notes

1 Vicon motion capture system: https://www.vicon.com/about-us/what-is-motion-capture/, accessed on 17 August 2021.

2 The CogentLabs dataset [17] includes data from 2 6D IMUs worn on the chest and thigh by 42 subjects. The activities recorded include a number of ADLs, five different types of falls, near falls, transitions between activities and ascending/descending stairs. The DLR dataset [18] includes data from 1 9D IMU worn on the belt by 19 subjects. Activities represented include 15 ADLs and unspecified types of falls. *We have failed to identify which ADLs and unspecified types of falls referred to by [12], the published dataset includes 12 ADLs.*

3 The ADLs performed in [42] include: stand-sit-stand, squat, sit-lie-sit and walk.

4 According to [13], the UMAFall dataset [19] contains data from 2 9D IMUs worn on the wrist and chest by 17 subjects. Activities recorded represent three types of falls (lateral, frontal and backward) and six ADLs (walking, bending, jogging, running, sitting and lying) [19]. *We note that [19] also contains data from two additional 9D IMUs worn on the ankle and waist and four additional ADLs, information on [13]’s selection of data is lacking.*

5 The 15 fall-like activities in [44] consisted of: sitting, lying, bending and squatting in sequence.

6 The fall-like activities used as near-falls in [46] were: slip, trip, being hit/bumped, misstep and S2S.

7 The ADLs performed in [46] were: walk 5 m, sit down on a chair after standing, lie down on floor after standing, rise up to standing from chair and pick up object from floor.

8 The ADLs performed in [47] were: sit down and stand up, do a squat and stand up, walk 5 m turn around and walk back and lie down and get up from bed. The fall types performed were: sideways on left and right side respectively, backward and forward.

9 The UniMiB Shar [23] dataset includes data from a smartphone accelerometer worn in a trouser pocket by 30 subjects (18-60 years old). Activities recorded include nine different ADLs and eight types of falls. Only a portion of the ADL data was used in [15]. The other dataset [24] includes data from an RFID-tag with an embedded accelerometer worn on the sternum while performing a number of ADLs. *Interestingly, ref. [24] included 14 healthy older adults. Considering that age is an important factor [2,3,4], it is unclear why this information was left out in [15].*

10 The PAs conducted in [54] were: lie, sit, walk and jog.

11 The postures used in [56] were: sitting, walking, crawling, laying on all sides and kneeling.

12 The two sets of sensors used in [56] were 11 “Class-act” accelerometers positioned symmetrically on the calves, thighs, upper and lower arms, chest and on the right hip and ankle and five “Shimmer” accelerometers positioned on the right calf, thigh, upper and lower arm and chest.

13 The PAs performed in [57] were: walk, run and sit on a chair.

14 The PAs performed in [58] were: running, walking, brisk-walking and jumping jacks.

15 The PAs reported in [59] were: lying down, sitting down, slouching, standing, walking, stalling, running, indoor cycling and outdoor cycling.

16 Dataset 1 [20] includes data from five 9D IMUs worn by eight subjects on different body locations. Dataset 2 [21] includes data from five IMUs (*no dimension provided*) worn by four subjects on back, right/left lower arm and right/left upper arm. *We note that [21] includes 24 custom acceleration and gyroscope sensors distributed over the body. Further, it includes 12 subjects but [14] reported on only four of them, information on [14]’s selection was lacking.* Dataset 3 [22] includes data from five IMUs (3D accelerometer and 2D gyroscope) worn by three subjects on left wrist, right arm, left thigh, right ankle and waist. Each subject performed 4–12 PAs. *We note that [22] includes an additional IMU on the right wrist, information on [14]’s selection is lacking.*

17 The PAs performed in [61] were: 40 m walking, ascending a 10 step stair and descending a 10 step stair.

18 The PAs performed in [62] were: eat, errands, exercise, hobby, hygiene, sleep, travel, work and other.

19 Dataset 1 (PAMAP2 Physical Activity Monitoring Data Set [25]) includes data from three accelerometers and HR worn by subjects performing a series of nine PAs performed for <3 min with 1-min breaks in between. Inactive states, e.g., sitting, and high activity states, e.g., running, were included. *We note that sensors locations were not reported by [16] even though such information was provided in [25]*. Dataset 2 [26] includes data from an accelerometer worn by 15 subjects performing seven PAs ranging from computer work to walking in a similar data collection protocol. *We note that [26] reports only on 10 subjects, it is unclear why [16] reports on five additional subjects.*

20 The motor tasks performed in [67] were: S2S, putting on sock, putting lotion on the hands, buttoning shirts and drinking from a bottle.

21
*We note that the author lists of [67,68] are partly the same.*

22 The rehabilitation exercises performed in [70] were: (1) abduction—moving the leg laterally and away from body on the transverse plane and back again, (2) extension—moving the leg behind body on the sagittal plane and back again, (3) S2S, (4) gait and (5) bipodal bridge laying on flat surface with soles flat down and raising hip until there is a straight line between hip and shoulder and lowering hip down again; the transverse and sagittal planes are illustrated in Figure 1.

23 The PAs performed in [87] were: lying, sitting, standing, walking, wheeling (in a wheelchair) and ascending stairs.

24
*The LA results were retrieved using the same 24 PD patients but presented in [98], an article not retrieved in this literature search.*

25 The PAs performed in [100] were: standing, walking, running and cycling.

## Appendix A

**Table A1:** Information on demographics and study characteristics for included articles based on datasets.

Ref.	Source	No. of Participants	Age Group	Age Statistics	Male/Female	Patient/Healthy	Sensors	Assessment
[12]	Dataset [17]	42	-	-	36/6	-	2 6D IMUs on chest and thigh	fall detection
	Dataset [18]	19	-	-	11/8	-	1 9D IMU on a belt	
[13]	Dataset [19]	17	-	-	-	-	2 9D IMUs on the wrist and chest	risk categorization, discriminate between 3 fall types and 6 ADLs
[14]	Dataset: [20]	8	20–30	-	4/4	-	5 9D IMUs at different body locations	automatic detection of dynamic sensor contexts and activation of different expert models
	Dataset: [21]	4	-	-	-	-	5 IMUs (*dimension not provided* on the back, right/left upper arm and lower arm	
	Dataset: [22]	3	-	-	-	-	5 IMUs (3D accelerometer 2D gyroscope) on the waist, wrists, right arm, left thigh, right ankle	
[15]	Dataset [23]	30	18–60	-	-	-	1 smartphone 3D accelerometer in the trouser pocket	detect bed exits
	Dataset [24]	-	elderly	-	-	-	1 RFID containing 3D accelerometer on the sternum	
[16]	Dataset [25]	-	-	-	-	-	3 accelerometers, HR (*location not provided*)	PA life logging
	Dataset [26]	15	-	-	-	-	1 accelerometer on the chest	

**Table A2:** Information on demographics and study characteristics provided in the datasets. * = information in source differs from information presented within the dataset, the numbers presented are from the dataset.

Ref.	Source	No. of Participants	Age Group	Age Statistics	Male/Female	Patient/Healthy	Sensors	Activities
[17]	Ojetola 2015	32	18–51	24.9 ± 6.19	28/4	0/32	2 6D IMUs on the chest/thigh	14 fall types, 6 near-fall types
		10	18–51	23.9 ± 3.4	8/2	0/10	2 6D IMUs on the chest/thigh	stair transition
[18]	Frank 2014	19*	23–52*	-	11/8*	-	1 9D IMU on a belt	12 ADLs*
[19]	Casilari 2016	17	14–55	-	11/6	0/17	4 9D IMUs on the left wrist, waist, chest and ankle	10 ADLs, 3 fall types
[20]	Altun 2010	8	20–30	-	4/4	0/8	5 9D IMUs on the chest, wrists and outside of the knees	19 activities
[21]	Roggen 2010	12	-	-	-	-	24 custom acceleration and gyroscope sensors distributed over the body	2 series with many activities
[22]	Ghasemzadeh 2015	3	25–35	-	3/0	0/3	6 3D accelerometer 2D gyroscope on the waist, wrists, right arm, left thigh, right ankle	14 translational movements
[23]	Micucchi 2017	30	18–60	27 ± 12	6/24	0/30	1 smartphone 3D accelerometer in a trouser pocket	9 ADLs, 8 fall types
[24]	Wickramasinghe 2017	14	66–86	-	4/10	0/14	1 RFID 3D accelerometer on the sternum	lying, sitting and getting out of bed, sitting and getting up from chair, walking between bed, chair and door
[25]	Reiss 2012	9	-	27.22 ± 3.31	8/1	-	3 9D IMUs on the chest and dominant wrist/ankle, HR on chest	12 activities
[26]	Casale 2012	10	27–35	-	7/3	-	1 accelerometer on the chest	5 activities
[27]	Saleh 2021	15	21–53	32	8/7	-	3 9D IMUs and barometer on the chest, waist and wrist	44 ADLs, 35 fall types
